# Germline organization in *Strongyloides* nematodes reveals alternative differentiation and regulation mechanisms

**DOI:** 10.1007/s00412-015-0562-5

**Published:** 2015-12-12

**Authors:** Arpita Kulkarni, James W. Lightfoot, Adrian Streit

**Affiliations:** Department Evolutionary Biology, Max Planck Institute for Developmental Biology, D-72076 Tübingen, Germany

**Keywords:** Germline, Nematodes, *Strongyloides*, Histone modification, Germline chromatin

## Abstract

Nematodes of the genus *Strongyloides* are important parasites of vertebrates including man. Currently, little is known about their germline organization or reproductive biology and how this influences their parasitic life strategies. Here, we analyze the structure of the germline in several *Strongyloides* and closely related species and uncover striking differences in the development, germline organization, and fluid dynamics compared to the model organism *Caenorhabditis elegans*. With a focus on *Strongyloides ratti*, we reveal that the proliferation of germ cells is restricted to early and mid-larval development, thus limiting the number of progeny. In order to understand key germline events (specifically germ cell progression and the transcriptional status of the germline), we monitored conserved histone modifications, in particular H3Pser10 and H3K4me3. The evolutionary significance of these events is subsequently highlighted through comparisons with six other nematode species, revealing underlying complexities and variations in the development of the germline among nematodes.

**Electronic supplementary material:**

The online version of this article (doi:10.1007/s00412-015-0562-5) contains supplementary material, which is available to authorized users.

## Introduction

**Fig. 1:**
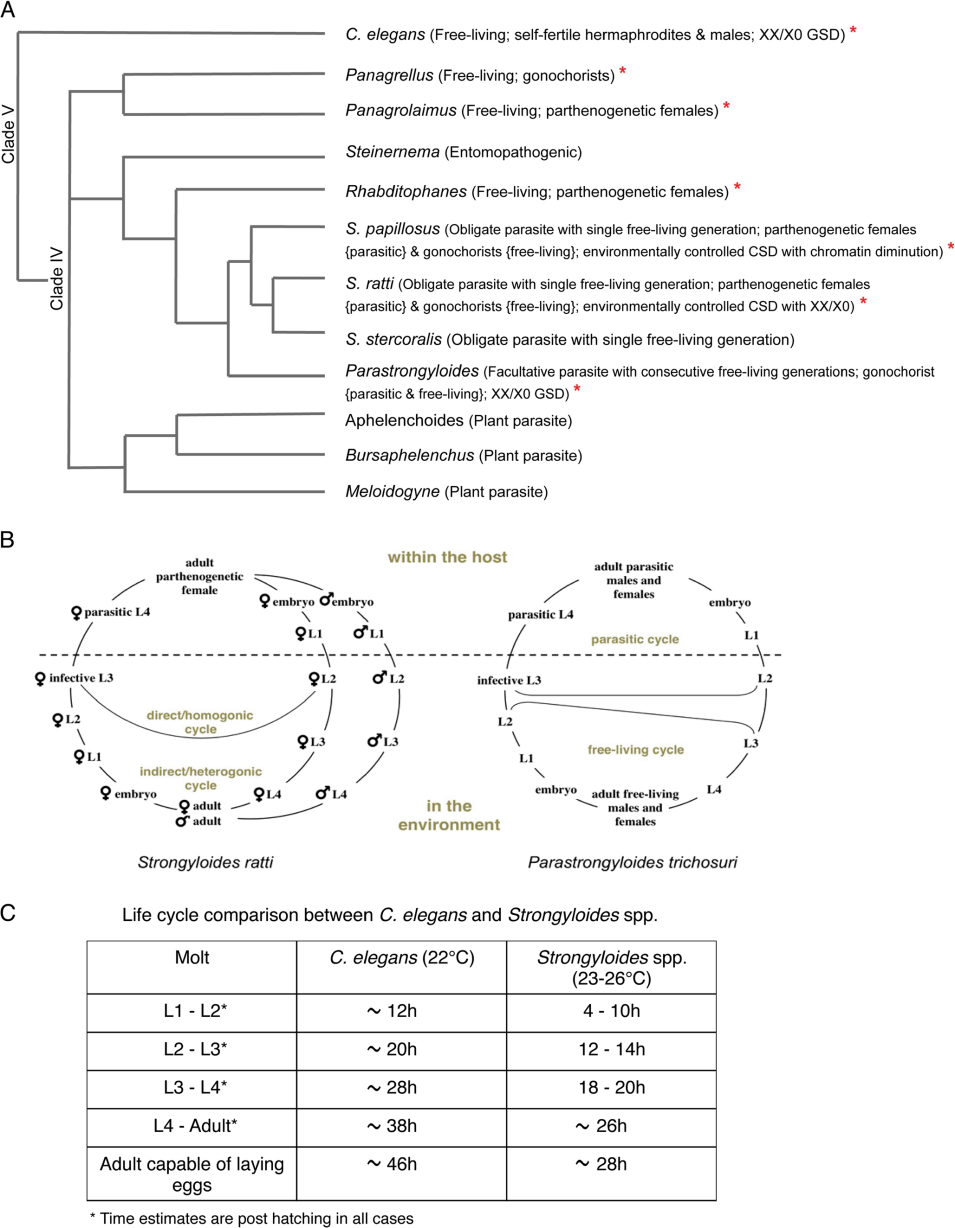
Introduction to *Stronygloides* nematodes. **a** A schematic cladogram to illustrate the phylogenetic position and interrelationships of *Strongyloides* with other nematode species based on Holterman et al. (2006). Species discussed in the text are marked with *red asterisks*. In *brackets* are the lifestyles and for the relevant species, their modes of reproduction (*GSD* genetic sex determination, *CSD* chromosomal sex determination, *XX/X0* sex determining system where females have two X-chromosomes and males only one). Branch lengths on this cladogram are irrelevant. **b** The generalized life cycle of *Strongyloides* species (*left*) compared to the life cycle of *Parastrongyloides trichosuri* (*right*). In the text infective L3 larvae are abbreviated as L3i. **c** A comparison of the developmental timing between *C. elegans* (according to WormAtlas) and *Strongyloides* spp. from hatching (for *C. elegans*) or start of culture (deposition of feces by the host for *Strongyloides* spp.) to adulthood. For *Strongyloides* spp., molt times are in a wide range because not all embryos/larvae are of exactly the same age in freshly deposited feces and additionally due to the fact that males develop and molt faster in comparison to females

Roundworms of the genus *Strongyloides* are widespread small intestinal parasites of various vertebrates (Viney and Lok 2015). Several members of this genus are being developed as model organisms and are particularly useful for parasitological research (of medical and veterinary interest) and for the study of basic biological questions such as host parasite interactions (Bleay et al. 2007; Crook and Viney 2005; Viney et al. 2006) and evolution (Fenton et al. 2004; Gemmill et al. 2000; Streit 2014). Additionally, the genus *Strongyloides* is in an interesting position phylogenetically, having close relatives representing extremely divergent modes of reproduction and lifestyles, ranging from being free-living (further subclassified as facultative or obligate animal parasites) to entomopathogenic and even plant parasitic (Fig. 1a) (Blaxter et al. 1998; Holterman et al. 2006). Therefore, *Strongyloides* spp. and their relatives have great potential for further development as highly informative models for comparative evolutionary studies. Our current understanding of such evolutionary aspects comes from other nematode species like the well-established (but phylogenetically distantly related) nematode models *Caenorhabditis* and *Pristionchus* spp. (Sommer and Bumbarger 2012). The rat parasite *Strongyloides ratti* (*S. ratti*) and the sheep parasite *Strongyloides papillosus* (*S. papillosus*) have been developed as model representatives of this genus (Eberhardt et al. 2007; Viney 1999; Viney and Lok 2015). While *S. ratti* can be conveniently maintained in their natural host, *S. papillosus* is maintained in rabbits, which act as permissive laboratory hosts. The possibility of and ease in accessing free-living stages, in addition to a number of recently developed resources for working with *Strongyloides* spp. and their close relative *Parastrongyloides trichosuri* (*P. trichosuri*), a facultative parasite of Australian possums (Eberhardt et al. 2007; Grant et al. 2006a, b; Nemetschke et al. 2010b; Shao et al. 2012; Viney et al. 2002), render this group of parasites more experimentally utilizable. The nematode genera of *Strongyloides* and *Parastrongyloides* together constitute the family of Strongyloididae (Dorris et al. 2002).

### Life histories of *S. ratti*, *S. papillosus*, and *P. trichosuri*

The life cycle of *Strongyloides* spp. (Fig. 1b) has been reviewed recently (Streit 2008; Viney and Lok 2015). The parasitic worms are all female and live in the small intestines of their respective hosts. They reproduce by mitotic parthenogenesis but nevertheless give rise to female and male offspring. These young female offspring have two lifestyle choices: they either develop into filariform third-stage infective larvae (L3i) and upon infection of a new host develop into parasitic adults (termed direct or homogonic development), or they develop by a rhabditiform L3 stage, along with all the males, to finally give rise to a facultative sexually reproducing free-living generation (termed indirect or heterogonic development). Offspring of the free-living adults are all female and bound to develop into parasites, with very few known exceptions (Streit 2008; Yamada et al. 1991). *P. trichosuri* is the best-studied representative of *Parastrongyloides*, a genus closely related to *Strongyloides* (Dorris et al. 2002). These species also form parasitic and free-living generations of reproducing adults. Nevertheless, their life history (Fig. 1b) and reproductive modes differ from those of *Strongyloides* spp. in interesting ways (Grant et al. 2006b). Firstly, parasitic males exist in *Parastrongyloides* (Mackerras 1959) and reproduction in this generation is sexual (Grant et al. 2006b; Kulkarni et al. 2013). Secondly, free-living *Parastrongyloides* spp. produce progeny of both sexes, and lastly, members of this genus (*P. trichosuri* in particular) have been shown to undergo an unlimited number of consecutive free-living generations (Grant et al. 2006b), making it a facultative parasite. However, the life cycle of the free-living generation in both *Strongyloides* and *Parastrongyloides* is rather short, even in comparison to *C. elegans* (Fig. 1c).

### Sex determination

In all species of *Strongyloides* investigated thus far, the sex ratio of the progeny produced by parasitic females is under the influence of the host’s immune system (Streit 2008), with an increasing immune response against the worms leading to a higher proportion of males. Male and female *Strongyloides* worms normally differ in their chromosomes (Streit 2008). However, the finer details of the sex chromosomes may differ among species. For example, *S. ratti* females have two X-chromosomes, but males have only one, in addition to the two pairs of autosomes in both sexes. Hence, *S. ratti* employs an environmentally influenced XX/X0 sex determination with 2n = 6 in females and 2n = 5 in males (Harvey and Viney 2001; Nigon and Roman 1952). In *S. papillosus*, the genetic material homologous to *S. ratti* chromosomes X and I is combined into one large chromosome (Nemetschke et al. 2010a). In the males of this species, sex-specific chromatin diminution creates a hemizygous region largely corresponding in sequence to the X-chromosome in *S. ratti*. Presumably, this chromatin diminution event helps to functionally restore the ancestral XX/X0 sex determining system (Albertson et al. 1979; Kulkarni et al. 2013; Nemetschke et al. 2010a). During this process, an internal portion of one chromosome is eliminated but both ends are retained as separate chromosomes, leading to the 2n = 5 in male and 2n = 4 in female karyotypes. By contrast, *P. trichosuri* employs chromosomal XX/X0 sex determination with 2n = 6 in females and 2n = 5 in males. In this species, there is no indication for an environmental influence on sex determination (Grant et al. 2006b; Kulkarni et al. 2013).

### The germline in the nematode family of Strongyloididae

**Fig. 2:**
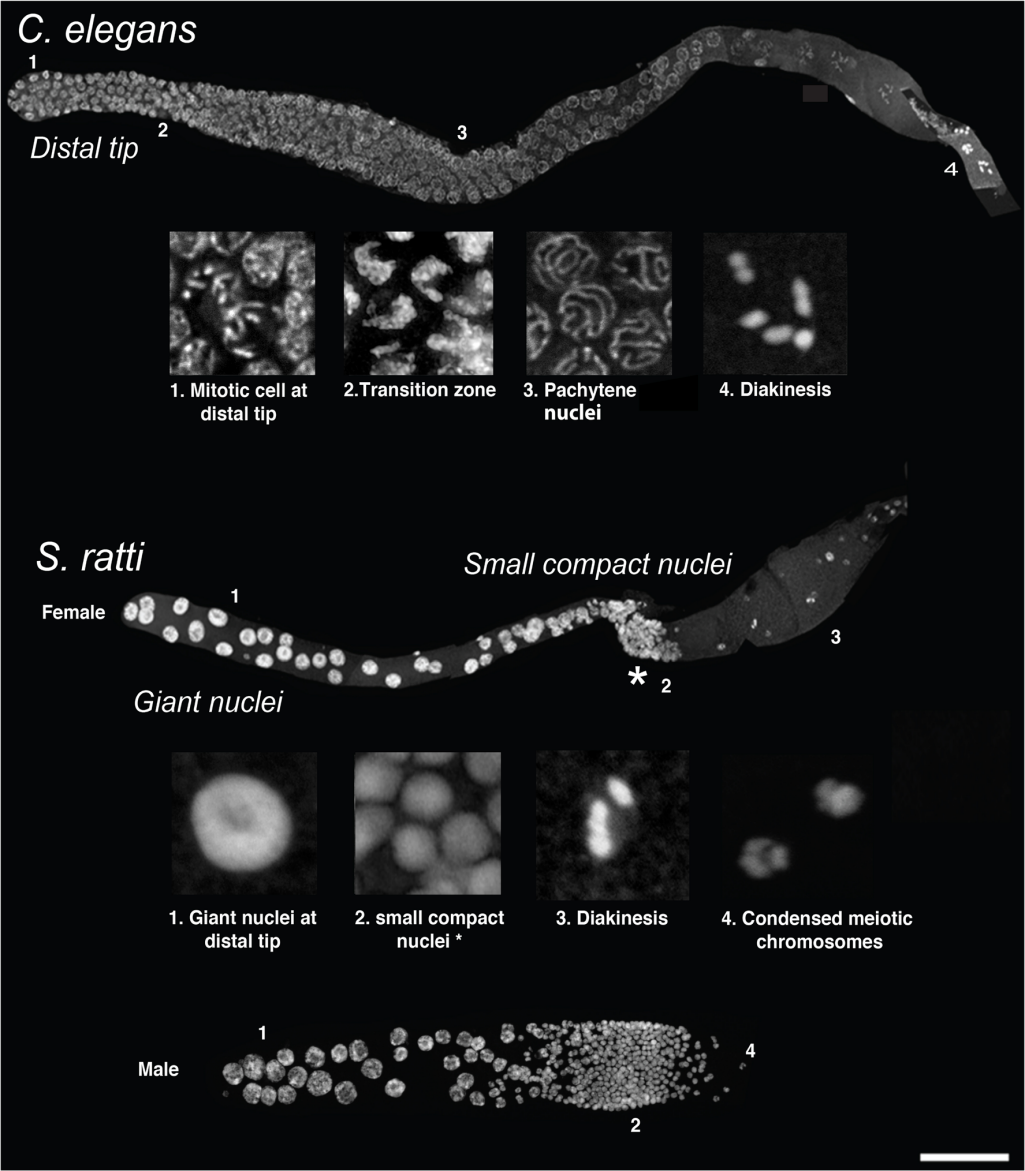
Comparisons between the *C. elegans* and *Strongyloides* gonads. *Top*: a DAPI-stained dissected *C. elegans* hermaphroditic (adult) gonad, showing progression of germ cells in the germline (distal tip is to the *left*). The *numbers 1–4* indicate the immediate insets below, with each inset showing the characteristic morphology (mitotically dividing cells at distal tip, crescent-shaped nuclei at transition zone, “bowl of spaghetti” in the pachytene zone and condensed chromosomes at diakinesis, respectively) of germ nuclei for those regions. *Bottom*: DAPI-stained dissected gonads from *S. ratti* adult females (*top*) and males (*bottom*) showing the completely different gonadal organization in comparison to *C. elegans*. Note the shorter but broad nature of the *S. ratti* male gonad in comparison to the female (adult males are smaller in size to adult females; adults are approximately 28–30 h post-culturing). Here, the entire distal arm is occupied by intensely staining giant nuclei, followed by a band of small compact nuclei at the gonadal loop (*asterisk*). Except for even more strongly condensed small nuclei in males, the organization is identical in both sexes. *Insets 1* and *2* are derived from female gonads. The band of small nuclei is followed proximally in females by nuclei, which might be in diakinesis (shown in *inset 3* taken from a female germline) and in males with condensed presumably meiotic chromosomes (*inset 4*, taken from a male germline). *Scale bar* 50 μm

The gonads in both sexes of the model nematode *C. elegans* are essentially tubular (Hubbard and Greenstein 2005). The hermaphroditic gonad has two arms, one extending anteriorly and one posteriorly, with both arms terminating in a central vulva. In the males, the gonad has just one arm with a posterior opening. A somatic cell, termed the distal tip cell (DTC) sits at the very tip of each gonad arm and signals the nearby germ cells to proliferate mitotically via Delta/Notch signaling (Kimble and Crittenden 2005). Once cells move out of the reach of the DTC signal, they exit the mitotic cell cycle and initiate meiosis, thereby beginning their differentiation into gametes. Hence, *C. elegans*, like many other nematodes (Rudel et al. 2005; Rudel and Sommer 2003), maintains a stem cell population at the distal end of each gonad arm and this creates a constant flow of increasingly differentiated germ cells from the distal tip to the proximal end (Fig. 2 (top), *C. elegans* germline and insets 1–4). In addition, all cells in the distal portion of the gonad, which is situated at the dorsal side of the adult worm, open into a common central rachis and form a large syncytium.

While the overall morphology of the gonad is very similar to that of *C. elegans*, the appearance and organization of the germ cells differ greatly in *S. ratti* and other members of the Strongyloididae (Fig. 2, *S. ratti* germlines and insets 1–4) to that of *C. elegans* (Hammond and Robinson 1994; Triantaphyllou and Moncol 1977). For *S. ratti*, the distal arm was shown to contain giant nuclei, which have been reported to have a DNA content of up to several hundred C (Fig. 2, *S. ratti* inset 1), with 1C being the DNA content of a haploid set of chromosomes (Hammond and Robinson 1994). The distal arm (i.e., the region with the giant nuclei) is followed by a band of very small, compact nuclei at the gonadal loop (Fig. 2, *S. ratti* inset 2), proximal to which presumably meiotic nuclei with condensed chromosomes can be observed (Fig. 2, *S. ratti* insets 3 and 4). Further along the gonad, differentiated oocytes or sperm (depending on the sex) are present, very similar to *C. elegans*.

Other than these basic morphological aspects, information regarding germline development and gene expression for Strongyloididae members is currently lacking. For *C. elegans*, many aspects of the control of germline development and gene expression have been shown to act through chromatin modifications, with these being extensively studied and well characterized (Kimble and Crittenden 2005; Schaner and Kelly 2006). Histone modifications have also been shown to be important determinants for establishing transcriptionally active or inactive domains in different organisms (Rando 2012), and therefore are an integral part of germline regulation.

Here, we present the first detailed comparative characterization of the germlines of free-living males and females of Strongyloididae (*S. ratti*, *S. papillosus*, and *P. trichosuri*) and compare this with *C. elegans*. We confirm that in the adult gonad, the distal arm is occupied by giant nondividing polyploid nuclei, and that there are no mitotically proliferating stem cells here, despite the presence of a DTC-like cell in these species. Additionally, it appears that proliferation of the germ nuclei is restricted to early and mid-larval development. Further, we describe differences in the germline chromatin of Strongyloididae members (especially in *S. ratti*) and *C. elegans*, by using conserved histone modifications with a particular emphasis on Histone3 phosphorylated at serine10 (H3Pser10) and Histone3 tri-methylated at lysine4 (H3K4me3). The variations in these histone modifications are further explored through comparisons with three other nematode species (*Rhabditophanes KR3021*, *Panagrolaimus PS1159*, and *Panagrellus PS1163*), each of which are at an increasing phylogenetic distance to *S. ratti*, unveiling further complexities in germline development among nematodes. Finally, we discuss the possible implications this has on regulating cell cycles and active transcription for Strongyloididae members.

## Materials and methods

### Culturing and manipulating nematodes

*S. ratti* ED321 and *S. papillosus* isolate LIN were maintained as described (Eberhardt et al. 2007; Nemetschke et al. 2010b; Viney et al. 1992). All animal experimentation was done according to national and international guidelines, with local authorities granting the required permits. *P. trichosuri* was cultured in continuous free-living cycles (Grant et al. 2006b) at 20 °C on NGM plates seeded with *Escherichia coli* (*E. coli)* OP 50 bacteria (Stiernagle 1999) supplemented with a piece of autoclaved rabbit feces. *C. elegans* N2, *Panagroliamus PS 1159*, *Panagrellus PS1163*, and *Rhabditophanes KR3021* were all maintained on NGM plates seeded with *E. coli* OP 50 bacteria at 20 °C, with the exception of *Rhabditophanes KR3021* which was kept at 15 °C.

### DAPI staining/microscopy

Adult worms (of the desired age) were fixed with ice-cold 100 % methanol (Roth GmbH and Co. KG) and directly mounted (without a rehydration series) on polylysine-coated glass slides in 10 μL of Vectashield (Vector Laboratories Inc., Burlingame, CA 94010) containing 1 μg mL^−1^ DAPI (4′,6-diamidino-2-phenylindole from Roche). Confocal stacks of the entire gonad were taken for manual counting of nuclei and processed in ImageJ (Fiji). For dissected gonad DAPI imaging, the same protocol was followed after gonad dissection.

### Gonad staging

Worms were collected by the Baermann funnel method every 2 h after hatching as described by Basir (1950). Each worm sample was split into two, one was directly viewed under the differential interference contrast (DIC) microscope and the other was used for DAPI staining and confocal imaging as described above.

### Transmission electron microscopy of *S. papillosus* gonads

Samples were cryo-fixed with a Baltec HPM-010 high-pressure freezer and were freeze-substituted in a Leica AFS-2 according to following protocol: 56 h at −90 °C in acetone with 5 % gallic acid monohydrate (Roth, Karlsruhe, Germany), 3 h warmed up to −60 °C, 5× washed with pre-chilled acetone, 24 h at −60 °C with 2 % OsO_4_, 0.5 % UA, 0.5 GA, 2 % H_2_0, warmed up to 0 °C at 4 °C h^−1^, 5× washed with pre-chilled acetone and infiltrated over a total period of 29 h with increasing concentrations of epoxy resin (EMbed-812-kit, Science Services, Munich, Germany); finally, the samples were cured at 60 °C for 48 h in flat embedding molds. Longitudinal semithin sections were stained with osmium tetraoxide and viewed in a FEI Tecnai G^2^ Spirit transmission electron microscope operating at 120 kV. Images were taken with a Gatan Ultrascan 4000 camera at maximum resolution using the manufacturer’s software.

### BrdU labeling of *C. elegans*, *S. ratti*, and *P. trichosuri* germlines

5-Bromo-2′-deoxyuridine (BrdU) labeling of germlines was done as described by Crittenden et al. (2006), and dissected germlines were visualized at different time points (3, 6, 12, and 16 h) by staining against an anti-BrdU-FITC-labeled antibody (BD Biosciences). For *Strongyloides* spp., males and females of various stages were manually selected and put on plates seeded with labeled *E. coli* and incubated for 3, 6, 12, or 16 h (in order to start the labeling with very young larvae, gravid females were allowed to lay their eggs on labeled *E. coli* plates). At the end of this time period, the larval stage the individuals had reached was scored and the germlines were dissected and used for antibody staining. Young *Strongyloides* larvae with prolonged exposure to labeled *E. coli* plates (<16–18-h exposure) were found to be visibly very sick or dead.

### Immunostaining of nematode species

Gonads from adult worms were dissected in egg buffer (118 mM NaCl, 48 mM KCl2, 2 mM CaCl2, 2 mM MgCl2, 5 mM HEPES) containing 0.1 % Tween and immediately fixed in 1 % paraformaldehyde for 5 min. Slides were frozen in liquid nitrogen, freeze-cracked, and then immersed for 1 min in methanol at −20 °C and transferred to PBS-Triton X-100 (1× PBS, 1.5 % Triton X-100). Blocking in 0.7 % BSA in PBS-Triton X-100 was then carried out for 1 h. Primary antibodies were incubated overnight at room temperature, slides were then washed three times for 10 min in PBS-Triton X-100, and secondary antibodies were added and incubated for 4–6 h at room temperature. Following three washes for 5 min in PBS-Triton X-100, the slides were counterstained in 10 μL of Vectashield (Vector Laboratories Inc.) containing 1 μg mL^−1^ DAPI (Roche). The following primary antibodies were used at the indicated dilutions: Anti-BrdU-FITC labeled (1:2.5, BD Biosciences), rabbit H3K9/K14ac (1:500, Diagenode), rabbit H4K20me1 (1:500, Diagenode), mouse H3K27ac (1:500, Diagenode), mouse H3K27me3 (1:500, Diagenode), rabbit H3K9me1 (1:500, Diagenode), rabbit H3Pser10 (1:200, Millipore), mouse H3K4me3 (1:500, Diagenode), and rabbit α-tubulin (1:200), rabbit α-Actin (1:200). The following secondary antibodies were used for visualization: Goat anti-rabbit labeled with Alexa Fluor 488 (1:200), goat anti-mouse labeled with Cy3 (1:200), and mouse anti-biotin labeled with Cy3 (1:200).

All images were acquired as a stack of optical sections with an interval of 0.65 μm using an Olympus confocal FV1000 microscope and processed in ImageJ (Fiji) and Adobe Photoshop CS.5.

### FISH in *S. ratti*

Gonads from adult worms were dissected in egg buffer (118 mM NaCl, 48 mM KCl2, 2 mM CaCl2, 2 mM MgCl2, 5 mM HEPES) containing 0.1 % Tween and immediately fixed in 1 % paraformaldehyde for 5 min. Slides were frozen in liquid nitrogen, freeze-cracked, and then immersed for 1 min in methanol at −20 °C and then transferred to 2× SSCT three times for 5 min each. Then, the slides were dehydrated for 3 min each in 70, 90, and 100 % ethanol after which the slides were left to air-dry. After air-drying, the slides were ready for adding the hybridization mix containing the florescent in situ hybridization (FISH) probes. The amount of probe per slide was between 150 and 200 ng. The final concentration of the hybridization mix after adding the probe was 2× SSCT, 50 % formamide, 10 % *w*/*v* dextran sulfate. Fifteen microliters of this mix was added per slide followed by heating the slides at 93 °C for 2 min in a heated block. The slides were then removed and incubated overnight at 37 °C in a humid chamber. For post-hybridization washes, the slides were rinsed in 2× SSCT 50 % formamide at 37 °C for 30 min. This was followed by three washes in 2× SSCT for 5 min each. Then, the slides were blocked in 1 % BSA in 2× SSCT for 30 min. Then, 50 μL of the primary H3Pser10 antibody was added per slide and incubated overnight at room temperature in a humid chamber. Then, slides were washed in 2× SSCT, three times for 10 min each, and incubated with 50 μL of the labeled secondary antibody (1:200) for 4–6 h. Finally, the slides were washed three times in 2× SSCT for 10 min each and were counterstained in 10 μL of Vectashield (Vector Lab. Inc.) containing 1 μg mL^−1^ DAPI (Roche) and ready for imaging using the Olympus confocal FV1000 microscope.

### Preparation of FISH probes

Probes (22–25 bp) were ordered from Eurofins MWG Operon (after ensuring preferably a single hit in the genome on the required chromosome). The ready-made probes were labeled internally with biotin along their entire length. They were visualized using a mouse monoclonal anti-biotin Cy3 antibody from Sigma-Aldrich.

**Table:** 

*S. ratti* FISH probe	Sequence	Chromosome and position	*ytP* marker/contig information
FISH-1	CGATCCATTCAAAAAGAAAGCTGAA	Autosome II at −4.1 cM	ytP91
FISH-2	CTGAACTTCAAGCAGAATTACGTGAAG	Autosome II at −4.1 cM	ytP91
FISH-3	CTTACTTTGGATAAATCATTTAA	Autosome II at 0.0 cM	ytP113
FISH-4	GAATATTGACCGTTGCTGGATCTTTA	Autosome I at 0.0 cM	ytP37
FISH-5	TTGCCGGAGTTCCGACAATGGGAG	Autosome I at 0.0 cM	ytP37
FISH-6	TGCTATGAAAGCTGGTTGGAAACAA	Autosome I at 2.2 cM	ytP117
FISH-7	ATTAAAATTACTGATAAATAACTC	Chromosome X	S.ratti_chrx_000001
FISH-8	AGCAGAATATAAAAGGAAGAACAAACTG	Chromosome X	S.ratti_chrx_000001
FISH-9	GTTATTTTCTATTAAAGACGGTGAAGA	Chromosome X	S.ratti_chrx_000001

## Results

### Timing of germ cell proliferation in Strongyloididae

**Fig. 3:**
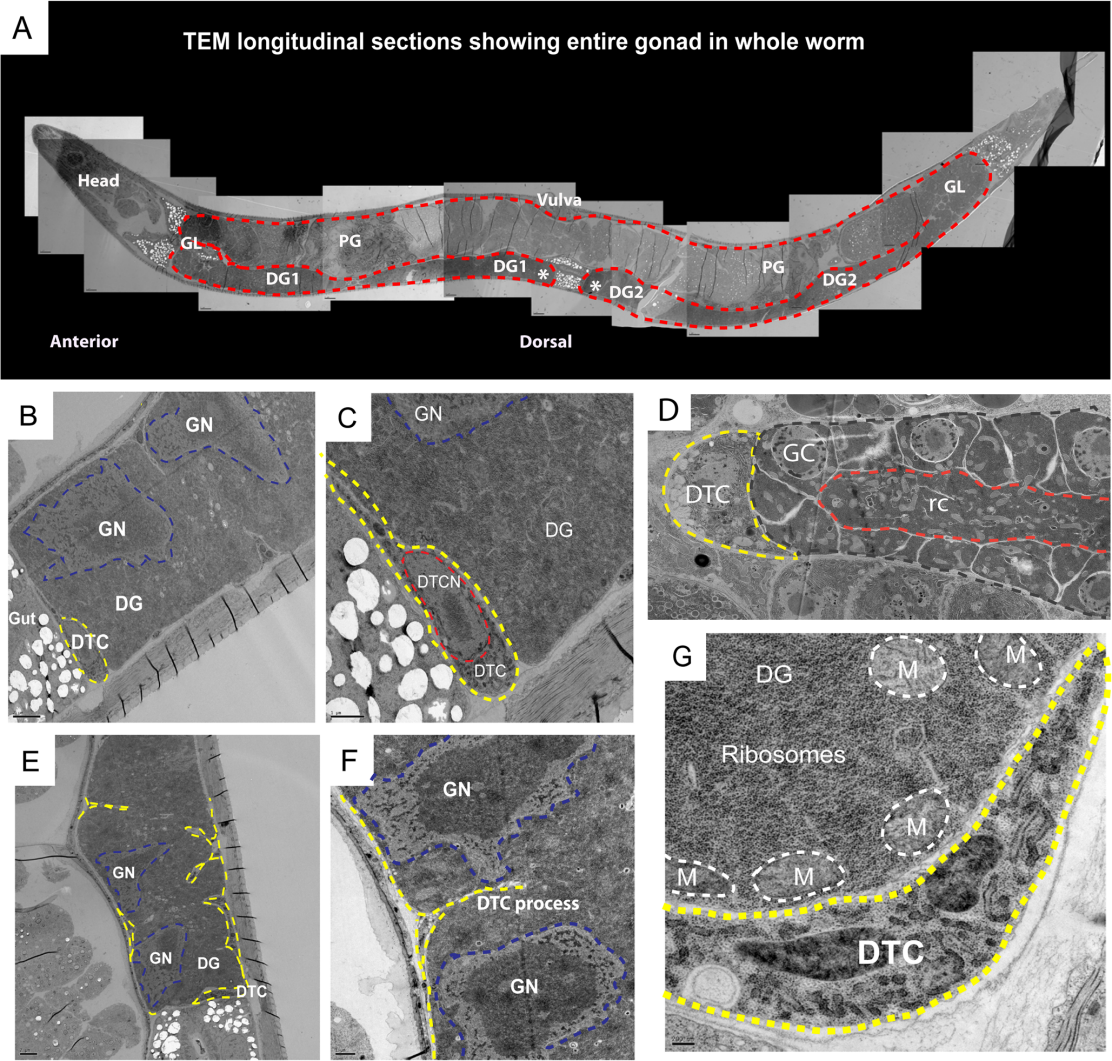
Transmission electron microscopy (TEM) of the *S. papillosus* free-living female gonad. **a** Semithin longitudinal TEM sections of a *S. papillosus* female showing the entire gonad (outlined in *red*) in the body of the adult worm, with the vulva (*top*, *central*). The two distal gonad arms are marked as DG1 and DG2 (distal tips are marked by *asterisks*); the gonadal loop is labeled as GL and the proximal gonad as PG. Female adults that were approximately 28–30 h post-fecal culturing were used for this analysis. **b** TEM section showing the distal tip of the gonad, *DG*, with giant nuclei, *GN* (outlined in *blue*), and the distal tip cell, *DTC* (in *yellow*). **c** Zoom in of the DTC (*yellow*) showing its nucleus, *DTCN* (in *red*) in addition to the giant nuclei, *GN* (in *blue*) in the distal gonad, *DG*. **d** TEM section from *Pristionchus pacificus* showing the DTC (outlined in *yellow*) sitting as a cap on the distal tip (outlined in *gray*), with germ cells GC, around a central rachis “rc” (in *red*). This organization is similar to what is found in *C. elegans* (image courtesy of Metta Riebesell). **e** The distal tip cell and its processes (outlined in *yellow*) making contact with the distal gonad at regular intervals. **f** Magnified view showing a DTC process (in *yellow*) contacting the distal gonad close to two giant nuclei GN (in *blue*). **g** Zoom in at the distal tip (with DTC outlined in *yellow*) showing the high density of ribosomes (electron dense regions) and mitochondria, *M* (outlined in *white*) in the distal gonad, *DG*

Simple cytological observation has revealed no obvious mitotic cell divisions in the germline of the adult free-living *Strongyloides* spp., implying that these organisms do not maintain a population of proliferating stem cells in their adult germlines. In order to test this hypothesis, we first performed a detailed electron and light microscopic characterization of the distal gonad of *S. papillosus* adult females (Fig. 3) and males (data not shown). Given the high degree of similarity in the organization of the gonads between *S. papillosus*, *S. ratti*, and *P. trichosuri*, we assume that this morphological description holds true for the latter species as well. Detailed cytological examination using the transmission electron microscope (TEM) found no indication for mitotic activity in any part of the germline, for either *S. papillosus* males or females. Nevertheless, a DTC was observed, which assumed a somewhat different position than in *C. elegans* or *Pristionchus pacificus* (Fig. 3b, c). Most notably, it appears that this DTC-like cell, which is noticeably smaller, may sit lop-sided in these species and does not necessarily cap the whole distal tip as it does in *C. elegans* or *P. pacificus* (Fig. 3d). We also observed processes of the DTC-like cell making contact internally with the gonad at regular intervals along the entire distal arm (Fig. 3e, f). Furthermore, we found the cytoplasm of the distal arm to be very rich in ribosomes and mitochondria (Fig. 3g).

**Fig. 4:**
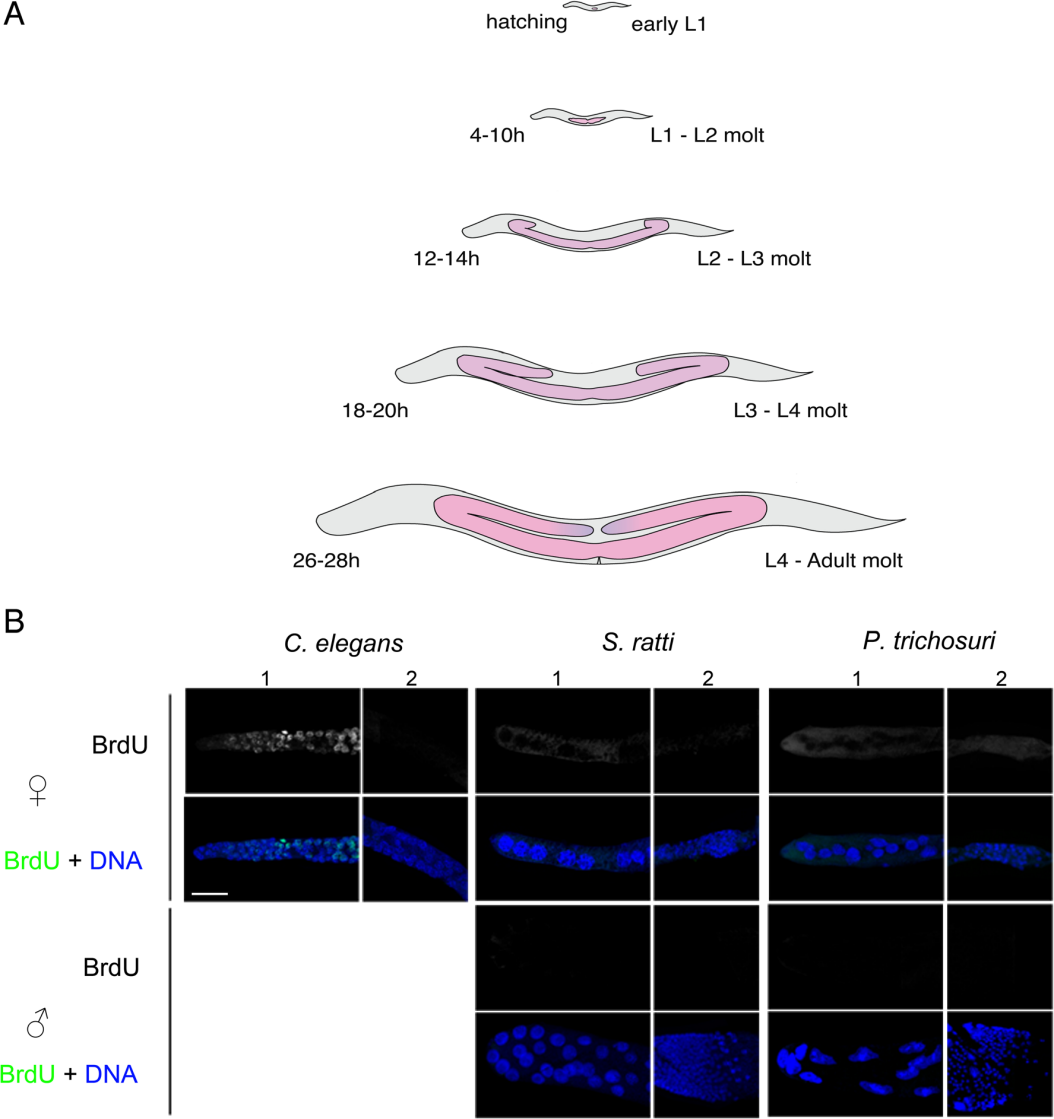
**a** Gonad size and shape at the time of the different molts. A cartoon representation of the worm is outlined in *gray* and the germline is represented in *pink* (with the distal tip in purple in L4 adults). The time post-feces deposition/culturing is indicated in hours to the left, with suitable approximations made for molt timings for the two *Strongyloides* species. The different larval molts are indicated to the *right*. For all stages anterior is to the right and ventral is below. **b** Bromo-deoxyuridine (BrdU) incorporation assays in *C. elegans*, *S. ratti*, and *P. trichosuri* adult germlines. *Panel 1* represents the distal gonad (with giant nuclei in *S. ratti* and *P. trichosuri*) and *panel 2* the gonadal loop region (with small nuclei in *S. ratti* and *P. trichosuri*). The top two rows show female (hermaphrodites in the case of *C. elegans*), and the two bottom rows show male germlines. The images were taken 3 h post-BrdU exposure. For both females and males, the BrdU channel is shown as separate in *gray* (*top*) or as merged with DNA (BrdU in *green*, DNA in *blue*). In *C. elegans* hermaphrodites, BrdU is seen incorporated into mitotically active cells in the proliferative zone at the distal tip of the germline 3 h post-BrdU exposure (*C. elegans*, *panel 1*). In comparison, no detectable BrdU uptake is seen within giant nuclei or the small compact nuclei in *S. ratti* and *P. trichosuri* (in either sex) indicating no active DNA replication in these regions in adults. Similar results were obtained at 6, 12, and 16 h post-exposure to BrdU in these two species, although a cytoplasmic signal was often seen. *Scale bar* 20 μm

In order to then determine when germ cell proliferation occurs and to ascertain the development of the germline at different larval stages, we stained different developmental stages of *S. papillosus*, *S. ratti*, and *P. trichosuri* with DAPI and additionally performed bromo-deoxyuridine (BrdU) incorporation experiments (BrdU uptake indicates actively cycling cells in S phase) for these species with *C. elegans* as a control. We first came up with a schematic for how the germline grows in these species, by correlating the development of the gonad at different larval molts under the differential interference contrast (DIC) microscope and with DAPI staining (Fig. 4a). In both species of *Strongyloides* and in *P. trichosuri*, we observed mitotic figures only in the germlines of larvae L3 and younger under DAPI staining (Suppl Fig. 1A). While such an in-depth analysis has not been carried out before, our results nevertheless corroborate earlier reports (Triantaphyllou and Moncol 1977), confirming no cellular divisions in the adult germlines of either sex. In addition, we observed that the giant nuclei first appeared in L3 larvae, coinciding with the arrest of all detectable mitotic germline activity in the distal arm. This is consistent with our observation of no BrdU in the adult giant or small nuclei, for animals that had been exposed to BrdU only after reaching the fourth larval stage (Fig. 4b). However, BrdU was incorporated into all the germline nuclei for animals that were exposed to it as younger larvae (SupplFig. 1B). BrdU was also readily detected in the germ nuclei of *C. elegans* control animals that were treated as L4 larvae and adults.

### Position of the central rachis and germline fluid dynamics in *S. papillosus* and *P. trichosuri*

**Fig. 5:**
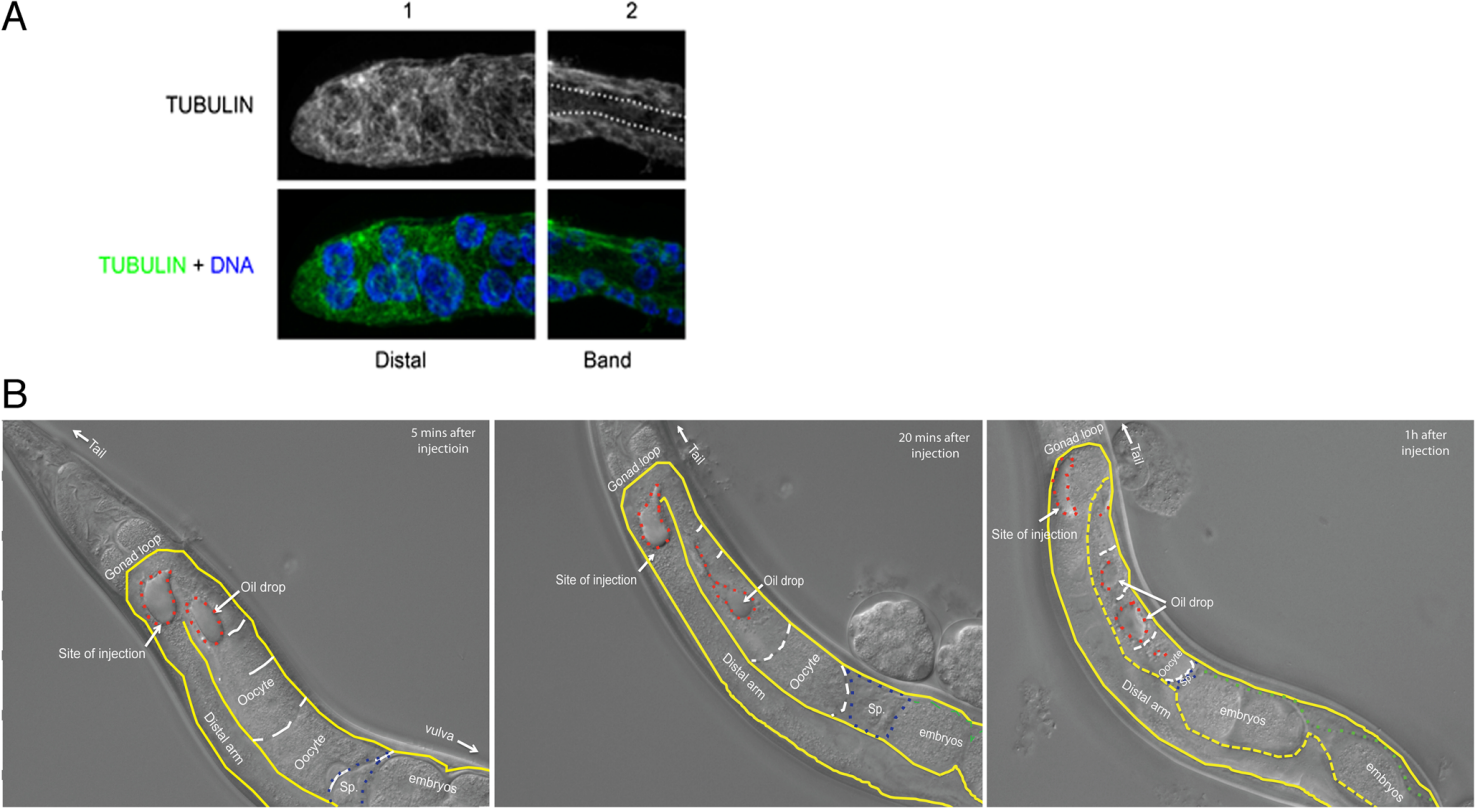
Central core and fluid dynamics. **a** The central core in *S. papillosus* dissected female gonads visualized by staining against α-tubulin (*green*) and DAPI (*blue*). *Panel 1* shows no visible central canal in the distal gonad (with the giant nuclei) when stained with α-tubulin (*top*), but a clearly visible canal in *panel 2* (outlined in *white*) starting just before the gonadal loop (beginning of the band of small nuclei). *Bottom panels* show merge with DAPI. **b** Differential interference contrast (DIC) time-lapse images of an injection experiment using mineral oil in a *P. trichosuri* adult female gonad, showing the rapid movement and incorporation of an oil droplet (if injected into the central core) proximally into growing oocytes. The gonad arm is outlined in *yellow*, the oil drop in *red*, developing oocytes are labeled and outlined in *white dashes*, the spermatheca (Sp.) is in *blue*, embryos labeled in the uterus outlined with *green*. Soon after injection, the single large oil drop is broken into smaller droplets, which quickly move proximally past the gonadal loop (seen in the left most image marked as the oil drop, at 5 min post-injection). Once proximal, the oil droplets move further down into developing oocytes (seen in the middle image at 20 min post-injection) to finally become incorporated within them (seen in the right most image, 1 h post-injection)

As the central rachis and syncytium is so prominent in the structure of the *C. elegans* germline (Hubbard and Greenstein 2005), we were interested in looking at the presence, position, and possible function of the central rachis in *Strongyloides* species. Using a combination of TEM and staining against α-tubulin (Fig. 5a), we found that the central rachis in these species begins just prior to the band of small compact nuclei before the gonadal loop region (in females), instead of running all along the distal arm as in *C. elegans*. In males, the rachis is slightly less pronounced, with it being detected at the base of the band of small compact nuclei (data not shown).

Due to the gonadal structure and function in *C. elegans*, there is a constant flow of cytoplasm from the distal tip toward the proximal end of the gonad, indicating active transport. If small lipid droplets are injected at any place in the distal gonad, they are transported proximally via the central rachis in the gonad, where-upon they are incorporated into growing oocytes (Wolke et al. 2007). As the central rachis in *Strongyloides* species was distinctively different in position, we performed the same experiment in *S. papillosus* and in *P. trichosuri* (Fig. 5b), obtaining in part similar results to *C. elegans*. While droplets placed just before the gonadal loop or at the loop moved proximally and were rapidly incorporated into growing oocytes, droplets injected along the distal arm were not transported at all in these species. This supports our microscopic observations (Fig. 5a) and confirms that a functional central rachis starts just prior to the gonad loop in these species.

### Meiotic progression and histone modifications in the germlines of *S. ratti*

**Fig. 6:**
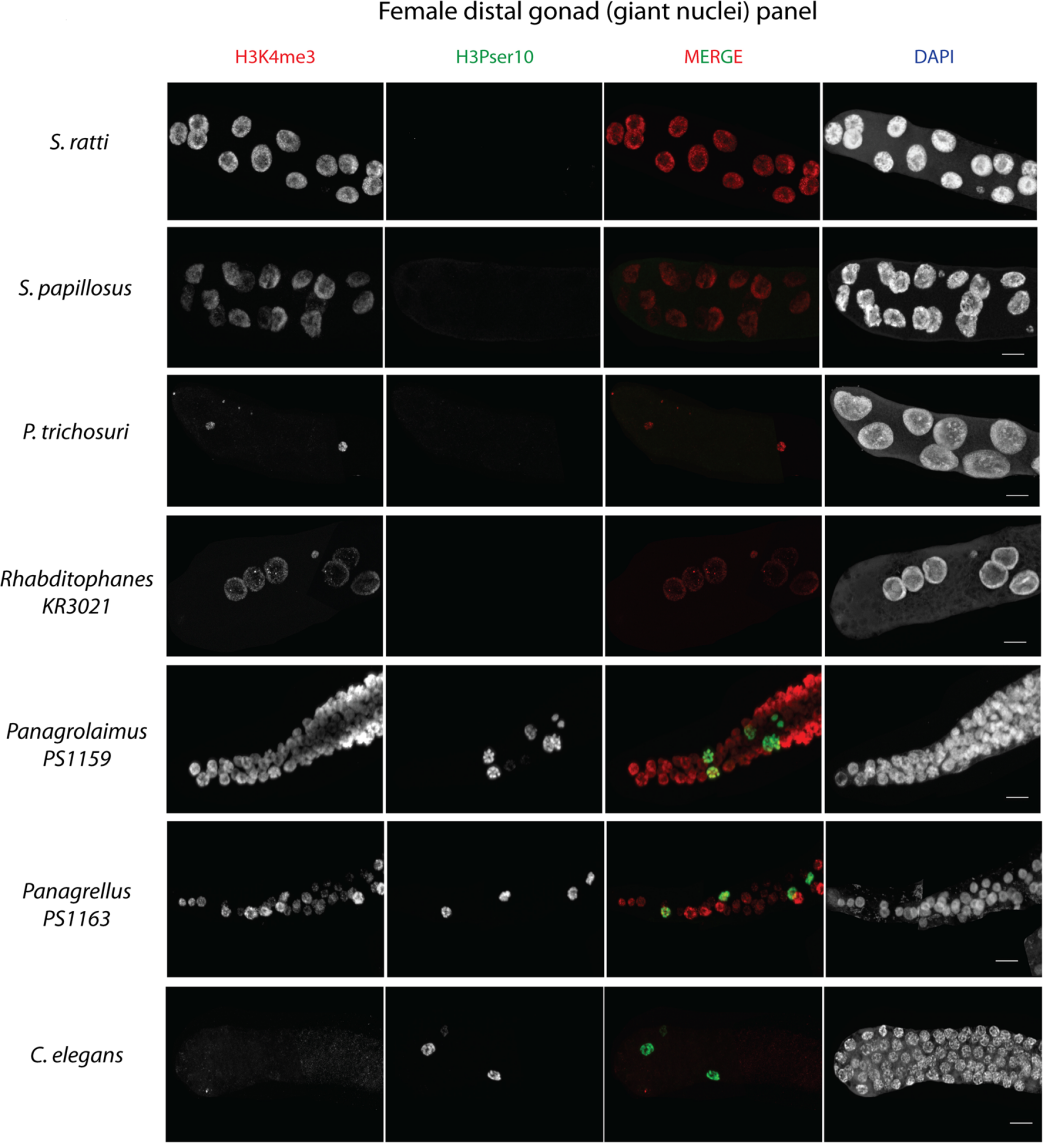
H3K4me3 and H3Pser10 staining patterns in females of different nematode species. H3K4me3 (in *red*) and H3Pser10 (in *green*) antibody stainings (with the individual channels separated according to color and labeled on top) in seven different (adult female) nematode species in the distal part of their gonads (distal tip is to the left for each). This region consists of giant nuclei in *Strongyloides* species. Note the similarity in gonad organization in *S. ratti*, *S. papillosus*, *P. trichosuri*, and *Rhabditophanes* KR3021, and between *Panagrolaimus PS1159*, *Panagrellus PS1163*, and *C. elegans*. For nematodes with a gonad organization similar to *S. ratti*, note the lack of H3Pser10 staining (in *green*) in the distal gonad, whereas its presence in species with a gonad organization similar to *C. elegans. Scale bar* 10 μm (note: adults are approximately 28–30 h post-culturing for *Strongyloides* species, but for other nematodes were young females carrying eggs in their uteri)

**Fig. 7:**
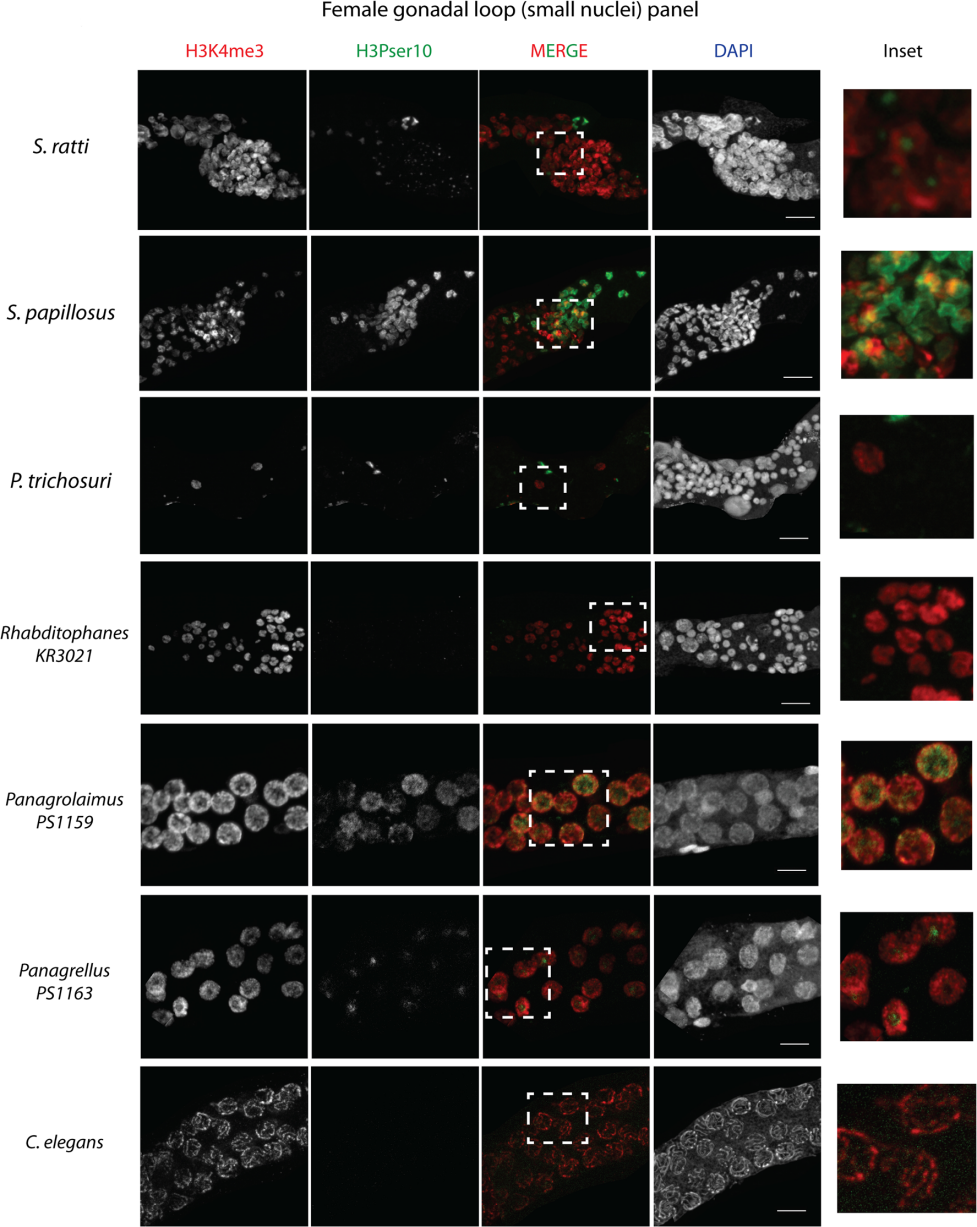
H3K4me3 and H3Pser10 staining patterns in females of different nematode species. A comparative image showing H3K4me3 (in *red*) and H3Pser10 (in *green*) staining patterns (individual channels are separated according to color and labeled on top) obtained in the same seven nematode species in the gonadal loop region (for orientation, distal gonad arm is to the left for each). This region consists of the small compact nuclei in *Strongyloides* species. *Insets* (extreme right) are magnified views of the indicated regions that are marked in white in the merge panels, showing co-localization patterns of H3K4me3 and H3Pser10 in each species. Note the localized dot-like H3Pser10 staining in the nuclei of *S. ratti* and *Panagrellus PS1163*, but its even distribution in the nuclei in *S. papillosus* and *Panagrolaimus PS1159*. H3Pser10 is absent in *P. trichosuri* (signal is from somatic sheath cells), *Rhabditophanes KR3021* and *C. elegans. Scale bar* 10 μm

Until now, nuclei in *Strongyloides* spp. were only recognized as meiotic once they had cytologically observable condensed bivalent chromosomes (Nigon and Roman 1952; Triantaphyllou and Moncol 1977). In an attempt to determine the actual position of the onset of meiosis in the *S. ratti* germline, we used antibodies that had in other systems (particularly in *C. elegans*) been shown to be meiosis specific. We stained dissected gonads of *S. papillosus* and *S. ratti* (both sexes) with anti-RAD-51 (Rinaldo et al. 2002) and anti-REC-8 (Pasierbek et al. 2003) antibodies, along with anti-SMC-3, which localizes to both meiotic and mitotic cells (Chan et al. 2003). In order to test if these commercially available antibodies might recognize the corresponding *Strongyloides* proteins, we also performed Western blot analyses. Although all three antibodies did recognize single defined bands of the expected size in Western blots (Suppl Fig. 2A), no meaningful immunostaining patterns were obtained with any of these antibodies in the *Strongyloides* germline (Suppl Fig. 2B, data shown for only *S. ratti* RAD-51 stainings). For the moment, it must remain open if RAD-51, REC-8, and SMC-3 are indeed not present in *Strongyloides* gonads or if the heterologous antibodies we used are not suitable to detect these proteins in situ for these species. In order to overcome this problem, we focused on antibodies against conserved histone modifications (Suppl Fig. 3A). We chose two histone modifications for comparison, one of which in *C. elegans* marks mitotically dividing germ nuclei (H3 phosphorylated at serine 10, H3PSer10) and the other marking transcriptionally active chromatin regions (H3 tri-methylated at lysine 4, H3K4me3), respectively (Hsu et al. 2000). Interestingly, while we did detect the published pattern in our *C. elegans* control (Hsu et al. 2000), H3Pser10 was completely absent from the distal gonad arm in *S. ratti* females (Fig. 6, *S. ratti* and *C. elegans* panels). Nevertheless, it was detected in the band of small compact nuclei, in a diffused and weak pattern (Fig. 7, *S. ratti* and *C. elegans* panels). In comparison, a very strong H3K4me3 signal was seen in the giant nuclei along the entire distal arm and in the small compact nuclei in female *S. ratti*. Additionally, it appears that H3K4me3 assumes a subnuclear localization that was mutually exclusive to the H3Pser10 signal in the small compact nuclei (Fig. 7, *S. ratti* panel and inset). By contrast, in *C. elegans*, H3K4me3 was weak but detectable at the distal tip and fairly strong in pachytene nuclei in accordance to previous literature (Schaner and Kelly 2006).

### Evolutionary significance of histone modifications

The dramatic differences in the germline histone modifications observed between *S. ratti* and *C. elegans* may be either associated with the structural and functional differences in the germlines of these two species, or because of their large phylogenetic distance. In order to ascertain the evolutionary significance of these differences, we performed similar stainings using anti-H3Pser10 and anti-H3K4me3 on dissected gonads of five other clade IV nematode species (*S. papillosus*, *P. trichosuri*, *Rhabditophanes KR3021*, *Panagrolaimus PS1159*, and *Panagrellus PS1163*). These species differ in their gonad organization such that *S. papillosus*, *P. trichosuri*, and *Rhabditophanes KR3021* resemble the gonad organization in *S. ratti*, while *Panagrolaimus PS1159* and *Panagrellus PS1163* are similar to *C. elegans*. Additionally, these species are of varying phylogenetic distance to *S. ratti*, while being equally distant from the clade V nematode *C. elegans* (Figs. 6 and 7; for nematode phylogenies, see Blaxter et al. (1998) and Holterman et al. (2006). We limited our analysis to females because some of the species involved were parthenogens. Although we observed striking differences between the species we studied, we noticed some general trends in staining patterns (Figs. 6 and 7 and Table 1). If species shared a gonad organization resembling that of *S. ratti* (i.e., *S. papillosus*, *P. trichosuri*, and *Rhabditophanes KR3021*), then they did not show any H3Pser10 staining in the distal gonad (Fig. 6, top four panels). Conversely, the free-living clade IV members including *Panagrolaimus PS1159* and *Panagrellus PS1163*, which have an overall germline organization more comparable with *C. elegans*, showed H3Pser10 staining in what appeared to be dividing germ cells in the distal gonad, just like in *C. elegans* (Fig. 6, bottom three panels). On the other hand, strong H3K4me3 staining extended up to the distal tip of the gonads of all clade IV members analyzed, with the notable exception of *P. trichosur*i, which did not show either H3K4me3 or H3Pser10 staining in the distal gonad (the few signals obtained for both antibodies are from nuclei of the somatic gonad; also note that all images shown are projections and not individual focal planes). Three (*S. papillosus*, *Panagrolaimus*, and *Panagrellus*) of the five clade IV species showed H3Pser10 staining in the gonadal loop region, just like in *S. ratti* (Fig. 7). Additionally, while in *S. ratti* and *Panagrellus PS1163*, the H3Pser10 signal was concentrated in a small region and the H3K4me3 and H3Pser10 signals appeared mutually exclusive, in *S. papillosus* and *Panagrolaimus PS1159*, the areas occupied by H3Pser10 were larger and partially overlapping with H3K4me3 (Fig. 7, insets). We also noted that in *S. papillosus* H3Pser10 stains strongly only in the proximal part of the band of small nuclei (Fig. 7, *S. papillosus* panel and inset). Even though we used roughly age-matched worms (adult females) for these analyses, it must be noted that we did find indications in *S. papillosus* that staining patterns with H3Pser10 depended at least partially on age (Suppl Fig. 3B).

**Table 1:** H3Pser10 and H3K4me3 staining patterns in seven different nematode species

Nematode (female)	Phylogenetic distance *S. ratti*	Gonadal organization similar to *Stongyloides*	Nematode clade	Lifestyle/mode of reproduction	Distal arm		Gonadal loop	
					H3Pser10	H3K4me3	H3Pser10	H3K4me3
*S. ratti*	–	–	IV	Free-living gonochorist	Absent	Present (high)	Present diffusely (nuclear localization distinct from H3K4me3)	Present high
*S. papillosus*	Closest *Stongyloides* species	Yes	IV	Free-living gonochorist	Absent	Present (high)	Present (entire nucleus)	Present high
*P. trichosuri*	Closest outgroup species	Yes	IV	Free-living gonochorist	Absent	Absent	Absent	Absent
*Rhabditophanes KR3021*	Closest sister family “Alloionematoda”	Yes	IV	Free-living parthenogen	Absent	Present (high)	Absent	Present
*Panagrolaimus PS1159*	Panagrolaimidae member	No (similar to *C. elegans*)	IV	Free-living parthenogen	Present (mitotic cells)	Present	Present (meiotic cells)	Present
*Panagrellus PS1163*	Panagrolaimidae member	No (similar to *C. elegans*)	IV	Free-living gonochorist	Present (mitotic cells)	Present	Present (meiotic cells)	Present
*C. elegans*	Farthest species in analysis	No	V	Free-living hermaphrodite	Present (mitotic cells)	Present	Absent	Present

### Sex-specific histone modifications in Strongyloididae

**Fig. 8:**
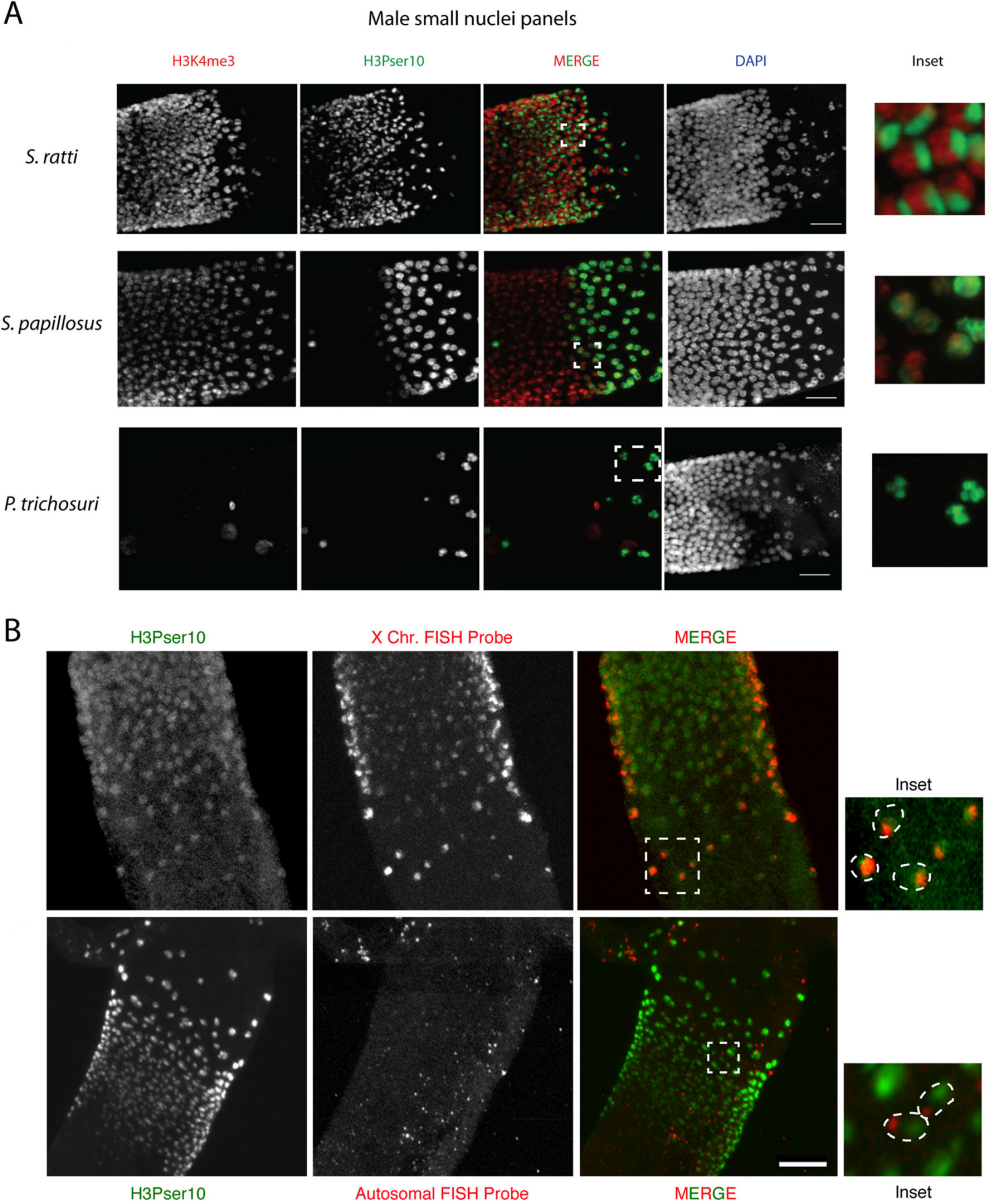
**a** A comparison of H3K4me3 and H3Pser10 staining patterns in the gonadal loop (containing small nuclei) of *S. ratti*, *S. papillosus*, and *P. trichosuri* males. *Insets* (*extreme right*) are magnified views of the areas marked in white in the merge panels, showing co-localization patterns with these two antibodies in each species. Note that the H3Pser10 staining is localized to a part of the nucleus in *S. ratti*, is evenly distributed in *S. papillosus* and found only on condensed meiotic chromosomes in *P. trichosuri. Scale bar* 10 μm. **b** FISH in *S. ratti* males. *Top*: the band of small nuclei in adult *S. ratti* males (for orientation, distal arm is to the top) showing co-localization of H3Pser10 (in green) and the X-chromosomal FISH probe (in red) within these nuclei. *Inset* (*extreme right*) is a magnified view of the region marked in white in the merge panel, with a rough outline of the nuclei (based on DAPI) marked as ovals using *dashed lines. Bottom*: the small nuclei in males show no co-localization (*inset*, *extreme right*) of H3Pser10 (in *green*) with an autosomal FISH probe (in *red*) within these nuclei. *Scale bar* 15 μm

In parallel, we investigated sex-specific histone modification differences within the Strongyloididae, by analyzing male gonads in *S. ratti*, *S. papillosus*, and *P. trichosuri* with H3Pser10 and H3K4me3 (Fig. 8a, Table 2). We found that the staining patterns in the distal gonads were indistinguishable between males and females of the same species (Fig. 6 and Suppl Fig. 2C). However, the small nuclei in males are far more condensed than in free-living females, allowing clearer visualization of H3Pser10 and H3K4me3 localization within the nucleus (Fig. 8a, insets). In *S. ratti* male small nuclei, the H3Pser10 signal occupied nearly a third of the nucleus and was distinct from H3K4me3, which occupied the remaining portion (see Fig. 8a, *S. ratti* inset). In *S. papillosus* males, H3Pser10 stained the entire nucleus evenly, with this staining being much stronger in the proximal part of the band of small nuclei (Fig. 8a, *S. papillosus* inset), just like our observation in *S. papillosus* females. In *P. trichosuri*, condensed spermatogenetic chromosomes proximal to the small nuclei stained strongly for H3Pser10, which was never observed in either of the two species of *Strongyloides* (Fig. 8a, *P. trichosuri* inset). Interestingly, in parasitic *S. ratti* and *S. papillosus* females, we found H3K4me3 and H3Pser10 staining patterns very similar to the ones in free-living males (Suppl Fig. 4). This is remarkable, given that parasitic females of *Strongyloides* are known to reproduce asexually by mitotic parthenogenesis and not undergo anything resembling meiosis.

**Table 2:** H3Pser10 and H3K4me3 staining patterns in Strongyloididae males

Nematode (male)	Phylogenetic distance to *S. ratti*	Gonadal organization similar to *Stongyloides*	Nematode clade	Lifestyle/mode of reproduction	Distal arm		Gonadal loop	
					H3Pser10	H3K4me3	H3Pser10	H3K4me3
*S. ratti*	–	–	IV	Free-living gonochorist	Absent	Present (high)	Present high (nuclear localization distinct from H3K4me3)	Present high
*S. papillosus*	Closest *Strongyloides* species	Yes	IV	Free-living gonochorist	Absent	Present (high)	Present high (entire nucleus)	Present high
*P. trichosuri*	Closest outgroup species	Yes	IV	Free-living gonochorist	Absent	Absent	Present (condensed meiotic chromosomes)	Absent

### H3Pser10 is an X-chromosome-specific histone modification in *S. ratti* males

In order to determine if the mutually exclusive regions staining for H3Pser10 and H3K4me3 represent different chromosomes, we performed FISH experiments with X-chromosome-specific and autosome-specific probes on dissected male *S. ratti* gonads (Fig. 8b). All our X-chromosome-specific FISH probes co-localized with the region of the nucleus displaying H3Pser10 staining (Fig 8b, top panel and inset). On the contrary, all autosome-specific probes did not co-localize with H3Pser10 staining (Fig 8b, bottom panel and inset). Therefore, at least in males, H3Pser10 appears to be an X-chromosome-specific histone modification in the small nuclei, indicating different chromatin modification states of the X-chromosome and the autosomes.

### Germline chromatin in *S. ratti*

Finally, in order to gain a better understanding of germline chromatin, and in particular chromosome-specific histone modifications, we analyzed five other histone modifications in the *S. ratti* germline, in both sexes (Suppl Figs. 5 and 6, Table 3). The five-histone modifications selected are associated with either active transcription, e.g., H3K9/K14ac and H3K27ac, or chromatin silencing, e.g., H3K9me1, H3K27me3, and H4K20me1 (Kouzarides 2007; Schaner and Kelly 2006). For evidence of chromosomal-specificity, we always co-stained with one of the two modifications (H3K4me3 or H3Pser10) described above. Antibodies against H3K9/K14ac and H3K27ac (SupplFig. 5) stained both the giant and the small nuclei in both sexes strongly, potentially indicating active transcription. This is consistent with our H3K4me3 stainings. In addition, we found indications for sex-specific staining differences for both these markers. In females, these two histone modifications stained the different gonad compartments evenly, while in the males, certain regions stained more brightly than others. In particular, H3K27ac stained giant nuclei at the distal tip more intensely than over the gonad arm (Suppl Fig. 5, H3K27ac male and female distal panels). Furthermore, H3K27ac stained small nuclei in the distal part of the band in males more strongly than toward the proximal (Suppl Fig. 5, male and female gonadal loop panels), whereas H3K9/K14ac stained small nuclei at the proximal part of the band more intensely than toward the distal (Suppl Fig. 5, male and female gonadal loop panels).

**Table 3:** Seven different histone modifications in *S. ratti* female germlines (A) and the same seven histone modifications in *S. ratti* male germlines (B)

A				
In female germlines				
Histone modifications	Distal arm (giant nuclei)	Band of small compact nuclei	Diakinesis	Chromosome specificity
H3^P^ser10	Absent	Present (very low)	Present	Yes—X chromosomal
H3K4me3	Present (high)	Present (high)	Present	Yes—autosomal
H3K9me	Present	Present (very low)	Absent	No—entire nucleus
H3K27me3	Present (punctuate)	Present	Present	Yes—autosomal
H3K9/K14ac	Present	Present (high)	Present	Yes—autosomal
H3K27ac	Present (high)	Present (high)	Absent	Yes—autosomal
H4K20me	Present	Present	Absent	No—entire nucleus
B				
In male germlines				
Histone modifications	Distal arm (giant nuclei)	Band of small compact nuclei	Spermatids	Chromosome specificity
H3^P^ser10	Absent	Present (high)	Absent	Yes—X chromosomal
H3K4me3	Present (high)	Present (high)	Absent	Yes—autosomal
H3K9me	Present	Present (very low)	Absent	No—entire nucleus, maybe more on X
H3K27me3	Present (punctuate)	Present	Absent	Yes—autosomal
H3K9/K14ac	Present	Present (high)	Absent	Yes—autosomal
H3K27ac	Present (high)	Present (high)	Absent	Yes—autosomal
H4K20me	Present (diffuse, high in some parts)	Present (high)	Absent	No—entire nucleus, stains X-chromosome intensely

Conversely, germlines of *S. ratti* stained against histone modifications known to be involved in silencing (H3K9me1, H3K27me3, and H4K20me1) showed only weak stainings (Suppl Fig. 6). In the giant nuclei of both males and females, H3K27me3 and H4K20me1 stainings could sometimes only be detected as bright puncta at the nuclear periphery, which is presumably stained heterochromatin (Suppl Fig. 6, distal gonad panels). Intriguingly, the only exception was H4K20me1, which stained the band region containing small nuclei in males very strongly (Suppl Fig. 6, male and female gonad loop panels). Overall, we observed similar staining patterns for all transcription activation markers used (namely H3K4me3, H3K9/K14ac, and H3K27ac), with all these modifications consistently staining both the giant and small nuclei. On the other hand, the silencing markers were absent or only weakly detected in these nuclei. The exact roles and functions of each of these histone modifications and their potential effect on germline development and regulation now need to be investigated further. Based on our results, H3Pser10 remains the only X-chromosome-specific histone modification identified in the *S. ratti* male germline. Consequently, it appears that *S. ratti* not only demonstrates an altered germline structure but also altered chromatin regulation through differing histone modifications in comparison to *C. elegans*.

## Discussion

Nematodes are powerful organisms in which to study the development and evolution of multiple organ systems. The genus *Strongyloides* consists of fairly close relatives displaying a wide range of lifestyles, with many species documented as economically relevant parasites of plants and animals (Blaxter et al. 1998; Holterman et al. 2006). However, a comparative approach for studying the evolution of the reproductive systems in such species has been lacking, compared to the well-studied but nonparasitic clade V nematodes like *C. elegans* and *P. pacificus* (Sommer and Bumbarger 2012). This work is therefore a first step toward bridging this gap using members of the genus *Strongyloides*.

Although *C. elegans* and *Strongyloides* nematodes share superficial morphological similarity and were classified as close relatives in the pre-molecular age (Blaxter et al. 1998), they differ greatly in many respects. One major difference is that the entire distal gonad of *Strongyloides* spp. consists of a population of endoduplicated giant nuclei, whereas the same region in *C. elegans* contains both proliferating germline stem cells and early meiotic nuclei. We, in agreement with earlier investigators (Hammond and Robinson 1994), have proposed that the polyploid giant nuclei in these parasitic nematodes might have nurse cell activity. Given the short reproductive window of *Strongyloides* nematodes (Gardner et al. 2004, 2006), these nuclei possibly provide a rapid supply of material during oogenesis, potentially allowing germ cells to move quickly through meiosis and early embryonic development. In *C. elegans*, there appears to be no specialized population of cells fulfilling this sole function. Instead, meiotic cells in pachytene (which go on to give rise to oocytes) are thought to perform this role (Wolke et al. 2007). All our results presented here are consistent with this proposed nurse cell function. This includes a distal arm rich in ribosomes and mitochondria (indicative of an active protein machinery) along with consistently high immuno-stainings in the giant nuclei for markers associated with transcriptional activity. Quantitative DNA and RNA sequencing of the distal gonad provided further evidence for high gene expression in the giant nuclei (Kulkarni et al. 2015). This study showed that autosomal genes are predominantly expressed in these nuclei (in comparison to X chromosomal ones), which is (at least partially) achieved by differential DNA amplification.

A second striking difference compared to *C. elegans* appears to be the lack of proliferating germline stem cells in the adult Strongyloididae gonad. None of our observations (i.e., our detailed light and electron microscopic analyses or the BrdU incorporation experiments) yield any indication of active DNA replication in adults. Although we have consistently observed H3Pser10 staining in the small nuclei at the gonadal loop of *Strongyloides* members, we think that this staining is not indicative of nuclear divisions in these species. This is a major difference compared to other systems including *C. elegans*, where H3Pser10 staining is directly indicative of cells in M-phase, never staining cells in S-phase (Hsu et al. 2000; Kadyk and Kimble 1998). Instead H3Pser10, for example in the case of *S. ratti*, is restricted only to a part of the nucleus and presumably a modification specific to the X-chromosome. Overall, we think that these observations together support the idea that Strongyloididae members build up a stock of germ cells during larval development, and then draw from this stock once they have matured. Indeed, free-living adults of these species are very short lived (one to two days of reproductive activity) and produce only a few dozen progeny (Gardner et al. 2004, 2006). For comparison, the longer-lived *C. elegans* can reproduce for several days and produce hundreds of progeny (Wegewitz et al. 2008).

Based upon its position at the distal tip, we have identified a somatic cell that may be the distal tip cell (DTC) in these species. The DTC in *C. elegans* has been implicated in maintaining a stem cell population in the distal gonad along with guiding the growth of the gonad arms during larval development (Kimble and Crittenden 2005). In this regard, it will be interesting to evaluate the developmental origin (lineage) of this DTC-like cell in *Strongyloides* spp., by performing ablation experiments, and then studying its role in gonad development, organization, and function.

Until the recent discovery of DNA methylation on N6-Adenine in *C. elegans* (Greer et al. 2015), it was thought that *C. elegans* lacks all DNA methylation (Simpson et al. 1986) and thus depends mainly on histone modifications to dictate chromatin structure. Histone modifications are thought to directly control function of local genomic regions, by increasing or decreasing the accessibility of DNA for transcription (Schaner and Kelly 2006). Since little is currently known about histone modifications in *Strongyloides* spp., to create an overview, we characterized multiple chromatin modifications of germ cells in *S. ratti*. We then focused on two modifications, namely H3K4me3 and H3Pser10 and used these for comparison with other clade IV nematodes and *C. elegans*.

In *C. elegans*, H3K4me3 brightly stains pachytene nuclei (in comparison to mitotic stem cells) known to be highly active transcriptionally (Schaner and Kelly 2006). The strong H3K4me3 stainings in the distal germlines (and at the distal tip) of *Strongyloides* and other clade IV nematodes (with the notable exception of *P. trichosuri*) might hint that the distal tip is a place of high transcriptional activity in these nematodes. The high presence of other transcriptional markers like H3K9/K14ac and H3K27ac at the distal tip (e.g., in *S. ratti*) provides further evidence for this. The absence of H3K4me3 in *P. trichosuri* is surprising and might possibly indicate that this modification is not a mark for active transcription in the giant nuclei of this species.

In the distal gonad of some clade IV nematodes (*Panagrellus* and *Panagrolaimus*) and *C. elegans*, H3Pser10 seems to be specific for dividing nuclei, implying that this role of H3Pser10 in the distal gonad may be conserved. In species without dividing cells in the distal gonad; we consistently did not observe any H3Pser10 staining in this part of the gonad. Surprisingly, in *S. ratti* males, H3Pser10 specifically marks a portion of the spermatogenic nuclei and based on the limited number of loci that we tested by FISH, this seems to be specific to the X-chromosome. Although some sperm without an X-chromosome (nullo-X sperm) exist in *S. ratti* (Kulkarni et al. 2015), it is likely that X-bearing sperm are preferentially made over nullo-X sperm, given that the males in this species sire only female progeny. Intriguingly, while H3Pser10 has been implicated in multiple gonad-related processes such as mitosis, apoptosis, and gametogenesis (Kouzarides 2007), it has also been implicated in dosage compensation (Wang et al. 2001), another process that requires distinguishing the X-chromosome from the autosomes. In this regard, we found two other histone modifications, namely H3K27me3 and H4K20me1 that are known to play a role in dosage compensation in other animals (Heard and Disteche 2006; Leeb and Wutz 2010; Payer and Lee 2008), to be abundant in the small compact nuclei of *S. ratti* males. This clearly indicates that further research is required to elucidate the functional consequences of these findings in these nematodes.

**Fig. 9:**
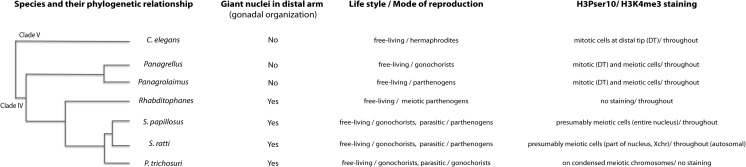
Phylogenetic relationships, gonadal organization, lifestyles, and histone modifications in the nematode species studied. Species with a gonad organization similar to *Strongyloides* (*Parastrongyloides* and *Rhabditophanes*) lack H3Pser10 staining in their distal arms, while species (*Panagrellus* and *Panagrolaimus*) with a gonad organization similar to *C. elegans* show H3Pser10 in this region

Interestingly, in *S. papillosus*, males lack an independent X-chromosome, and H3Pser10 was found to stain the entire nucleus evenly. For *P. trichosuri* (a species with an independent X-chromosome and known for producing male progeny), H3Pser10 marks both the condensed meiotic X-chromosomes and the autosomes alike. We never noted this modification on condensed meiotic chromosomes in either *S. ratti* or *S. papillosus* males. These striking and varied differences in H3K4me3 and H3Pser10 stainings within the three closely related representatives of the Strongyloididae suggests that this group of nematodes has either recently undergone, or is still in the process of undergoing rapid evolutionary changes. This in turn may be a response to becoming parasitic and/or parthenogenetic (in the parasitic generation). Overall, due to the current limitation in knowledge and tools for *Strongyloides* species, we must assume that the differences between *Strongyloides* spp. and *C. elegans* with regard to the different histone modifications are probably due to a combination of stochastic changes caused either by drift or because of adaptations that have accumulated over the time of their phylogenetic separation (Fig. 9).

It must be noted that the Strongyloididae gonad is probably a highly derived structure, and therefore, care must be taken while drawing direct conclusions based on other systems. First, one has to keep in mind that the gonads of clade IV nematodes are not as well characterized as in *C. elegans*, and so, it is not easy to define corresponding populations of cells. Another issue with *Strongyloides* spp. is that they need to be grown in fecal cultures, where the development of individual worms cannot be easily followed (the age of individual worms can therefore only be estimated). Nevertheless, from our comparative observations of the staining patterns with *C. elegans*, it appears likely that the small compact nuclei at the gonadal loop in *Strongyloides* spp. represent nuclei in some stage of meiotic development. If this is true then this small region is equivalent to the extended meiotic zone in *C. elegans* (occupying nearly two thirds of the distal arm length). This conclusion is supported by two observations: firstly, there is no other region in the gonad of these species which could be meiotic (given that this region is preceded by highly endoduplicated nuclei, and followed directly by oocytes or sperm) and secondly, the *Strongyloides* species we studied are known for their normal diploid genetics (Eberhardt et al. 2007; Nemetschke et al. 2010b; Viney et al. 1993), meaning that haploid gametes must necessarily be formed. It is of immense interest cytologically that the small nuclei look very different from meiotic cells of *C. elegans*. In particular, no condensed chromosomes reminiscent of the ones in the different meiotic zones from *C. elegans* appear in this region in members of Strongyloididae. Strikingly, these nuclei also transition from having noncondensed chromosomes to fully condensed bivalents rather abruptly, without any nuclei in intermediate stages of chromosomal condensation.

This study has opened up many questions of basic biological interest. In particular, we do not know if the different types of nuclei (the giant and the small nuclei) that we have described transform into one another over reproductive life. Due to current technological limitations with working on these species, all our observations so far have been limited to fixed specimens (this work and Kulkarni et al. 2015). Hopefully in the future, we will be able to observe the dynamics of these processes using marked chromatin, for example by visualization of GFP-tagged histone proteins.

Our findings presented here enhance our understanding of the biology of a group of fascinating but poorly understood parasites. Additionally, they also illustrate the usefulness of the *Rhabditophanes* spp. (free-living)/*Parastrongyloides* spp. (facultative parasite)/*Strongyloides* spp. (obligate parasite with a single free-living generation) in comparative studies. Given their multiple advantages, such systems could be used in the future to address questions about the emergence of parasitic lifestyles and the evolutionary consequences of becoming parasitic. Finally, novel insights into germline regulation during development may only be uncovered by looking into atypical species.

## Electronic supplementary material

Below is the link to the electronic supplementary material.

SupplFig. 1 **A**. DIC image (left) showing a *S. ratti* larva undergoing the L1 – L2 molt. The gonad is outlined in white in the body of the larva. The Inset (to the right) shows the corresponding DAPI staining (magnified view of the gonad) at L1 – L2 molt showing active proliferation of the germline. **B.** Dissected gonad from *S. ratti* L3-stage larva as control showing incorporation of BrdU in both giant and small nuclei (distal tip is to the left). Scale bar 10 μm. Notice that in gonads of worms exposed to BrdU as adults the BrdU signal is seen in the cytoplasm but excluded from the nuclei (cf Fig. 4B). (GIF 3722 kb)

High resolution image (TIF 24414 kb)

SupplFig. 2 **A.** Western blots for SMC-3, REC-8 and RAD-51 in *P. trichosuri*, *S. ratti* and *S. papillosus* adult worms (whole worm lysates) showing a single band at expected positions for each, with alpha-Tubulin used as loading control. **B**. RAD-51 staining (in green) in combination with H3K4me3 (in red) in the *S. ratti* male germline showing no RAD-51 in the distal gonad, the region with the giant nuclei (top) and no meaningful pattern in the band of small nuclei at the gonad loop (bottom). Much of the RAD-51 signal received here (shown in the inset, bottom right) is probably background noise, given that it was not in the same focal plane as the germ line nuclei (all images shown are projections of stacks from multiple focal planes). **C**. A comparison of H3K4me3 and H3Pser10 stainings in the distal gonads (region containing giant nuclei) in the adult males of *S. ratti*, *S. papillosus* and *P. trichosuri* (individual channels are separated according to color and labeled on top). Note the lack of H3Pser10 staining in *S. ratti* and *S. papillosus* in this region, but the presence of H3K4me3. For *P. trichosuri*, there is a complete lack of both H3K4me3 and H3Pser10 in this region. This pattern is similar to the stainings obtained in the females for each species. Scale bar 10 μm. (GIF 181 kb)

High resolution image (TIF 90110 kb)

SupplFig. 3 **A.** Western blots for H3Pser10 and H3K4me3 in *P. trichosuri*, *S. ratti* and *S. papillosus* adult worms (whole worm lysates) showing a single band at expected positions, with alpha-Tubulin used as loading control. **B.** H3Pser10 antibody staining of dissected young (bottom) and old (top) *S. papillosus* female gonads. The term ‘old’ is used here to indicate a mated female (or a female that has begun active oogenesis), whereas young is before the L4-adult molt. H3Pser10 is briefly seen on condensed chromosomes at the onset of mating, and from then on in the small nuclei, illustrating possible age related staining patterns in this species. (GIF 56 kb)

High resolution image (TIF 42887 kb)

SupplFig. 4 H3Pser10 and H3K4me3 staining in adult parasitic *S. ratti* and *S. papillosus* females. (**Top**) Distal and gonadal loop panels for *S. ratti* parasitic female. Inset (bottom) shows a zoom in marked in white in the merge panel. Note the mutually exclusive localization of H3Pser10 and H3K4me3. (**Bottom**) Distal and gonadal loop panels for *S. papillosus* parasitic female. Inset (bottom) shows a zoom in marked in white in the merge panel. Note the even localization of H3Pser10 and H3K4me3. (GIF 124 kb)

High resolution image (TIF 47892 kb)

SupplFig. 5 Histone modifications marking active transcription in the *S. ratti* germ line. **A**. Distal gonad panels (with distal tip to the left) from dissected male and female gonads of *S. ratti* adults stained against transcription activation markers H3K9/K14ac (top 2 panels) and H3K27ac (bottom 2 panels) in combination with H3K4me3 and H3Pser10 respectively. **B**. Corresponding gonadal loop panels for these males and females stained against the same antibodies. Scale bar 10 μm. (GIF 137 kb)

High resolution image (TIF 55882 kb)

SupplFig. 6 Histone modifications marking silencing in the *S. ratti* germ line. **A**. Distal gonad panels from dissected male and female gonads stained against H3K27me3 (top 2 panels), H3K9me1 (middle 2 panels) and H4K20me1 (bottom 2 panels) in combination with H3Pser10 or H3K4me3 respectively. **B**. Corresponding gonadal loop panels in these males and females for these same antibodies. Scale bar 10 μm. (GIF 218 kb)

High resolution image (TIF 90110 kb)
