# Supplementary figures and images for: Germline organization in Strongyloides nematodes reveals alternative differentiation and regulation mechanisms
Source: Chromosoma. 2015 Dec 12;125(4):725–45. doi: 10.1007/s00412-015-0562-5 (PMC5023735; doi:10.1007/s00412-015-0562-5)

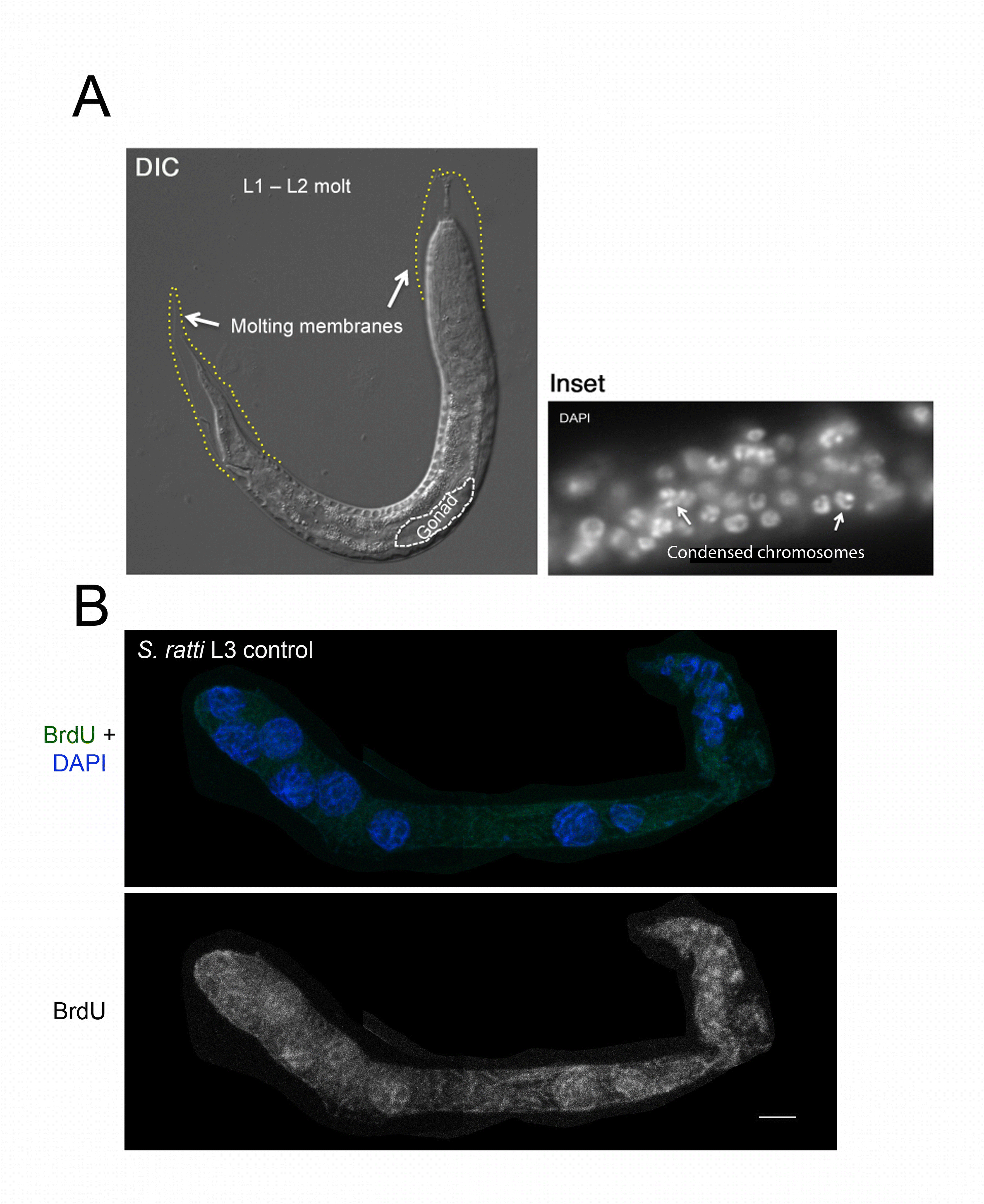

Supplement: Supplementary file 1 — A. DIC image (left) showing a S. ratti larva undergoing the L1 – L2 molt. The gonad is outlined in white in the body of the larva. The Inset (to the right) shows the corresponding DAPI staining (magnified view of the gonad) at L1 – L2 molt showing active proliferation of the germline. B. Dissected gonad from S. ratti L3-stage larva as control showing incorporation of BrdU in both giant and small nuclei (distal tip is to the left). Scale bar 10 μm. Notice that in gonads of worms exposed to BrdU as adults the BrdU signal is seen in the cytoplasm but excluded from the nuclei (cf Fig. 4B). (GIF 3722 kb) [file 412_2015_562_Fig10_ESM.gif]

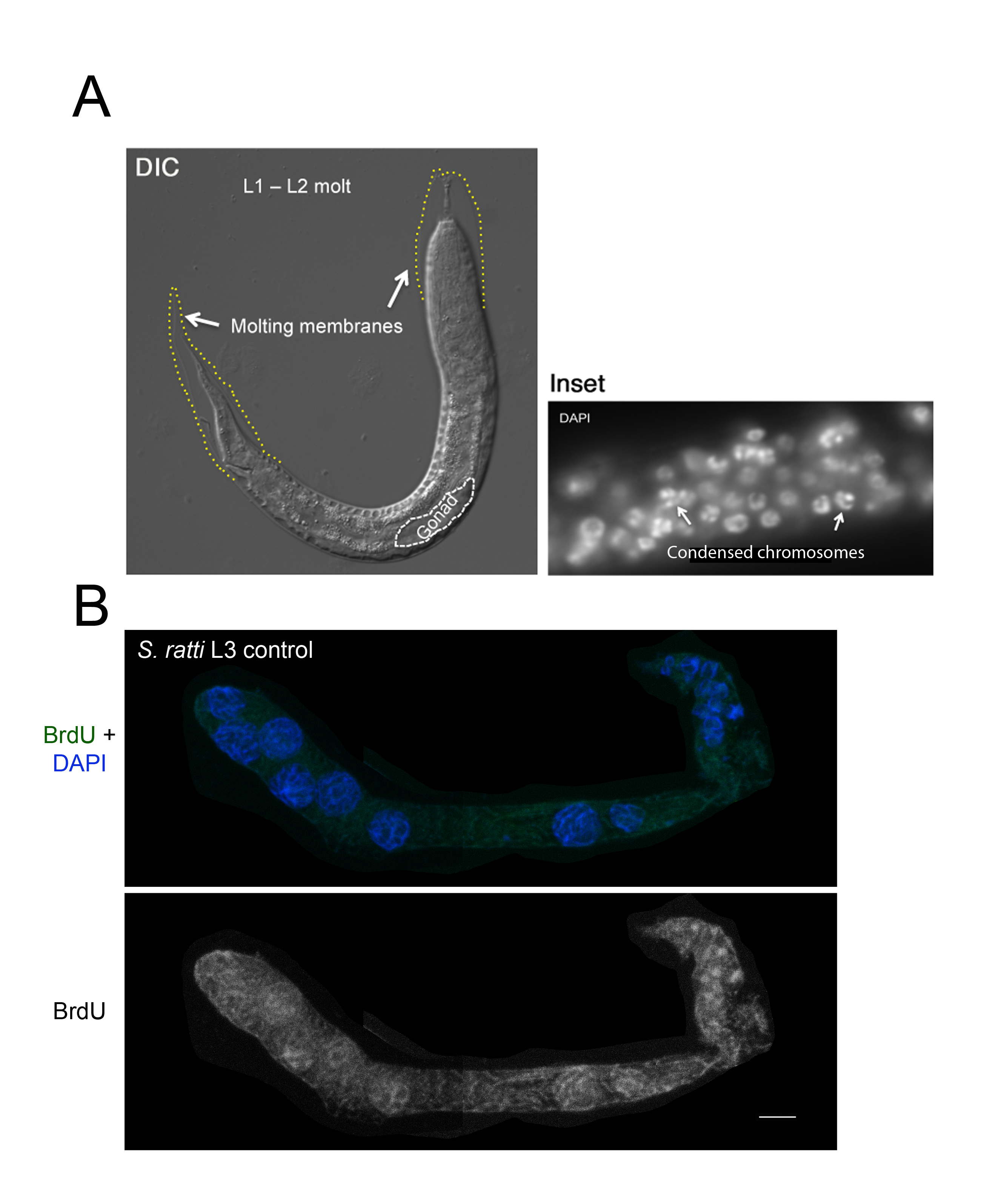

Supplement: Supplementary file 2 — High resolution image (TIF 24414 kb) [file 412_2015_562_MOESM1_ESM.tif]

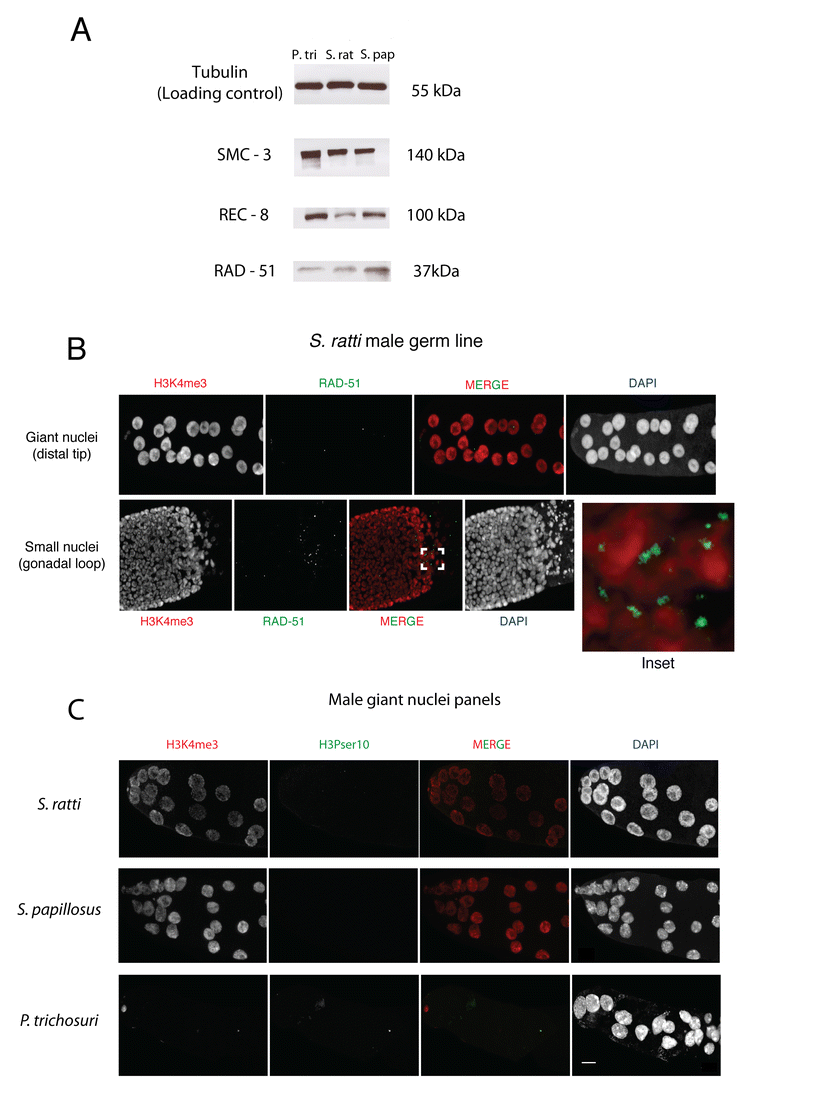

Supplement: Supplementary file 3 — A. Western blots for SMC-3, REC-8 and RAD-51 in P. trichosuri, S. ratti and S. papillosus adult worms (whole worm lysates) showing a single band at expected positions for each, with alpha-Tubulin used as loading control. B. RAD-51 staining (in green) in combination with H3K4me3 (in red) in the S. ratti male germline showing no RAD-51 in the distal gonad, the region with the giant nuclei (top) and no meaningful pattern in the band of small nuclei at the gonad loop (bottom). Much of the RAD-51 signal received here (shown in the inset, bottom right) is probably background noise, given that it was not in the same focal plane as the germ line nuclei (all images shown are projections of stacks from multiple focal planes). C. A comparison of H3K4me3 and H3Pser10 stainings in the distal gonads (region containing giant nuclei) in the adult males of S. ratti, S. papillosus and P. trichosuri (individual channels are separated according to color and labeled on top). Note the lack of H3Pser10 staining in S. ratti and S. papillosus in this region, but the presence of H3K4me3. For P. trichosuri, there is a complete lack of both H3K4me3 and H3Pser10 in this region. This pattern is similar to the stainings obtained in the females for each species. Scale bar 10 μm. (GIF 181 kb) [file 412_2015_562_Fig11_ESM.gif]

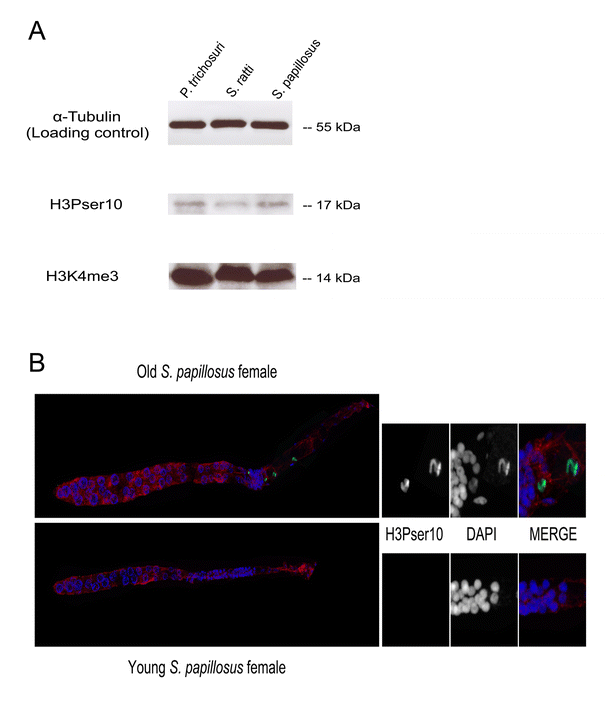

Supplement: Supplementary file 5 — A. Western blots for H3Pser10 and H3K4me3 in P. trichosuri, S. ratti and S. papillosus adult worms (whole worm lysates) showing a single band at expected positions, with alpha-Tubulin used as loading control. B. H3Pser10 antibody staining of dissected young (bottom) and old (top) S. papillosus female gonads. The term ‘old’ is used here to indicate a mated female (or a female that has begun active oogenesis), whereas young is before the L4-adult molt. H3Pser10 is briefly seen on condensed chromosomes at the onset of mating, and from then on in the small nuclei, illustrating possible age related staining patterns in this species. (GIF 56 kb) [file 412_2015_562_Fig12_ESM.gif]

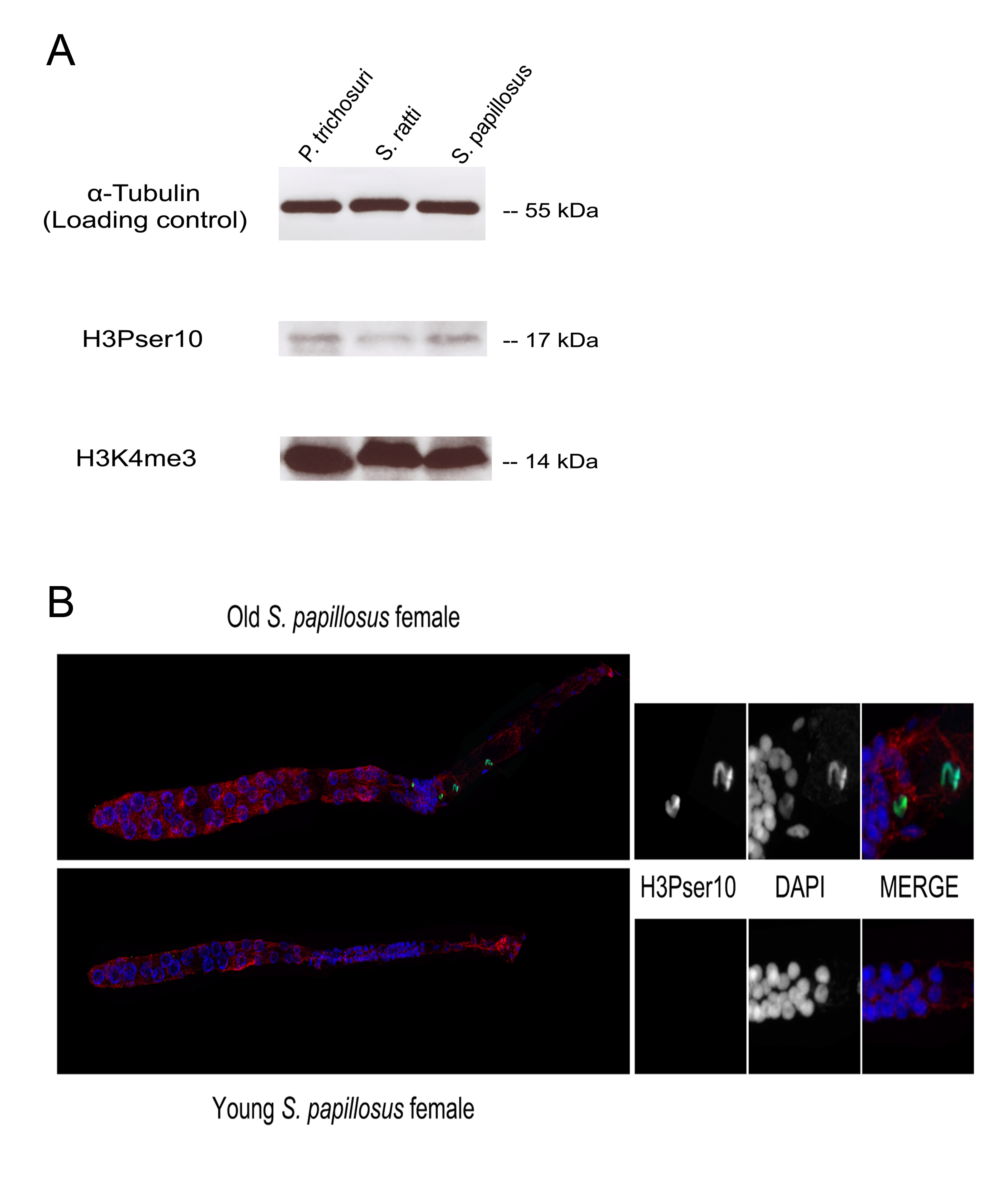

Supplement: Supplementary file 6 — High resolution image (TIF 42887 kb) [file 412_2015_562_MOESM3_ESM.tif]

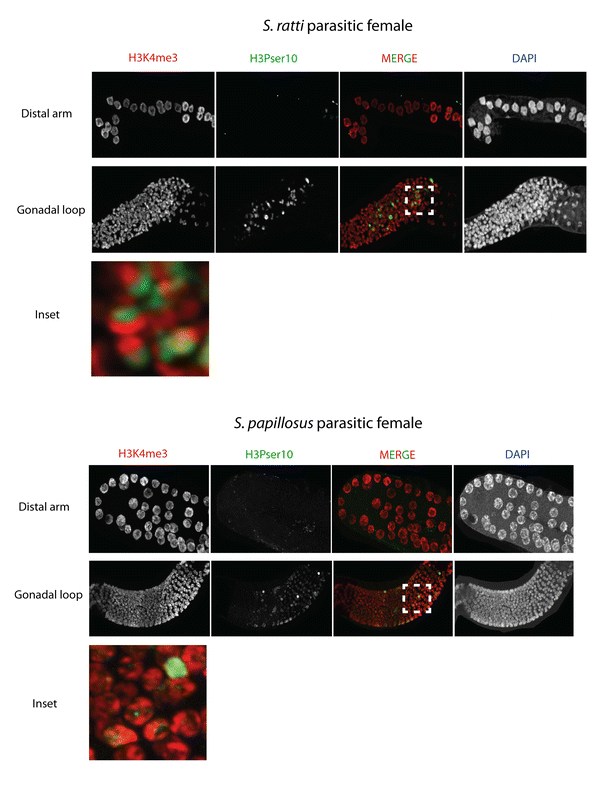

Supplement: Supplementary file 7 — H3Pser10 and H3K4me3 staining in adult parasitic S. ratti and S. papillosus females. (Top) Distal and gonadal loop panels for S. ratti parasitic female. Inset (bottom) shows a zoom in marked in white in the merge panel. Note the mutually exclusive localization of H3Pser10 and H3K4me3. (Bottom) Distal and gonadal loop panels for S. papillosus parasitic female. Inset (bottom) shows a zoom in marked in white in the merge panel. Note the even localization of H3Pser10 and H3K4me3. (GIF 124 kb) [file 412_2015_562_Fig13_ESM.gif]

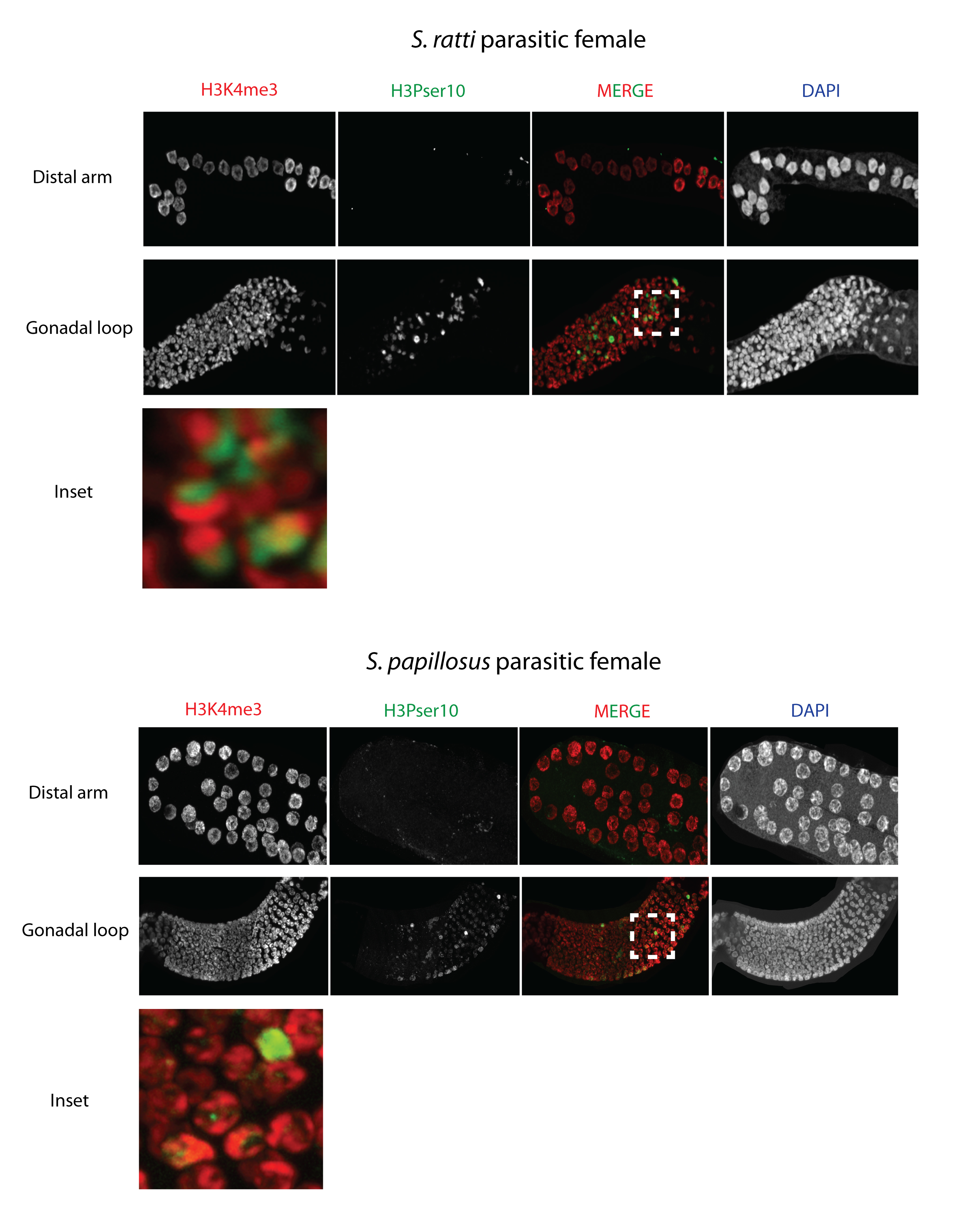

Supplement: Supplementary file 8 — High resolution image (TIF 47892 kb) [file 412_2015_562_MOESM4_ESM.tif]

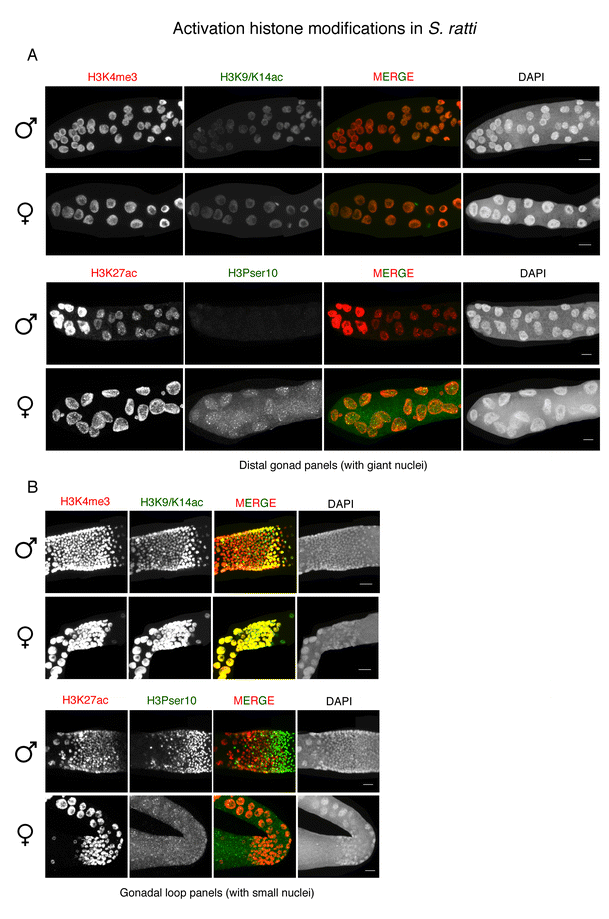

Supplement: Supplementary file 9 — Histone modifications marking active transcription in the S. ratti germ line. A. Distal gonad panels (with distal tip to the left) from dissected male and female gonads of S. ratti adults stained against transcription activation markers H3K9/K14ac (top 2 panels) and H3K27ac (bottom 2 panels) in combination with H3K4me3 and H3Pser10 respectively. B. Corresponding gonadal loop panels for these males and females stained against the same antibodies. Scale bar 10 μm. (GIF 137 kb) [file 412_2015_562_Fig14_ESM.gif]

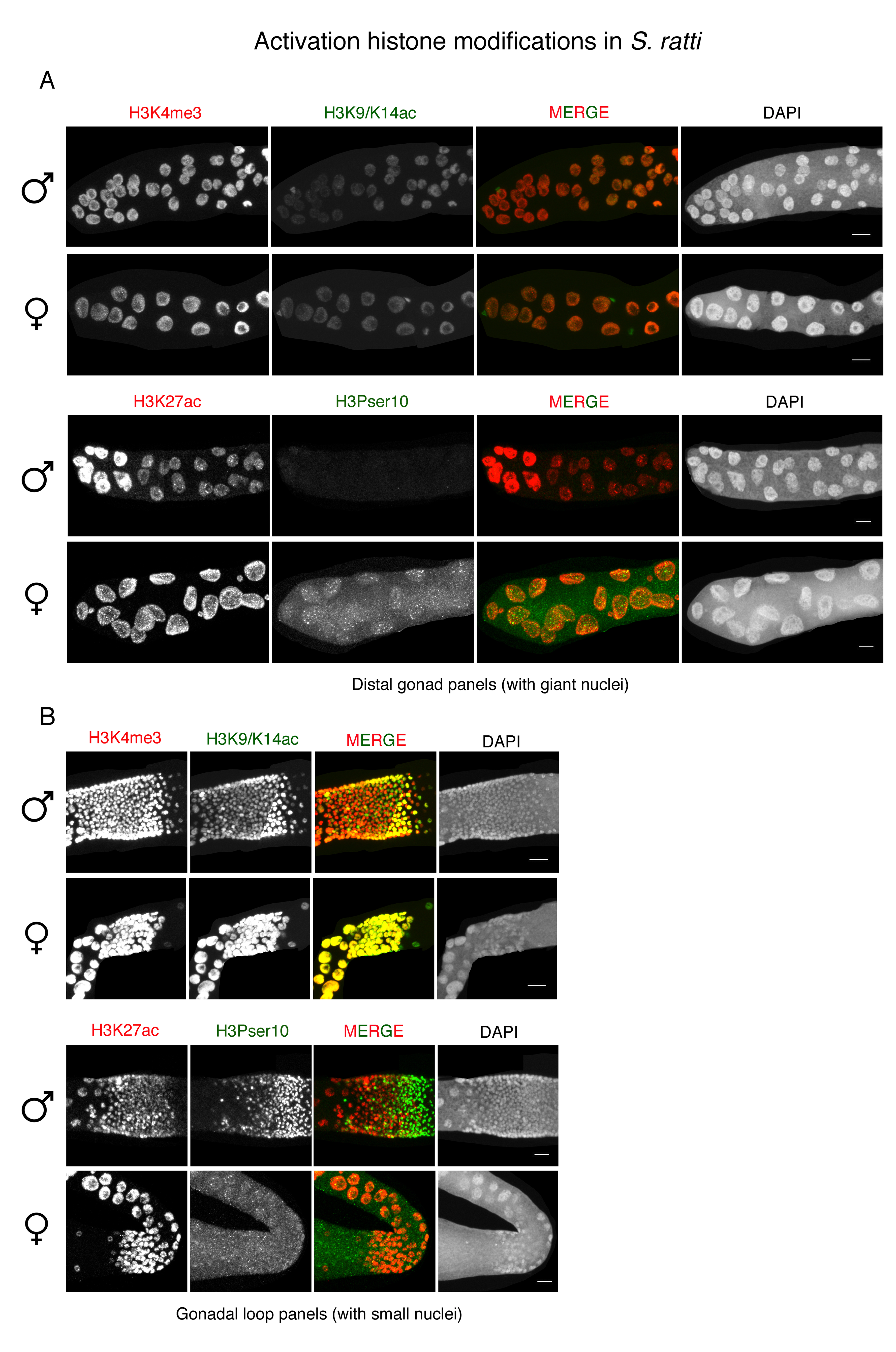

Supplement: Supplementary file 10 — High resolution image (TIF 55882 kb) [file 412_2015_562_MOESM5_ESM.tif]

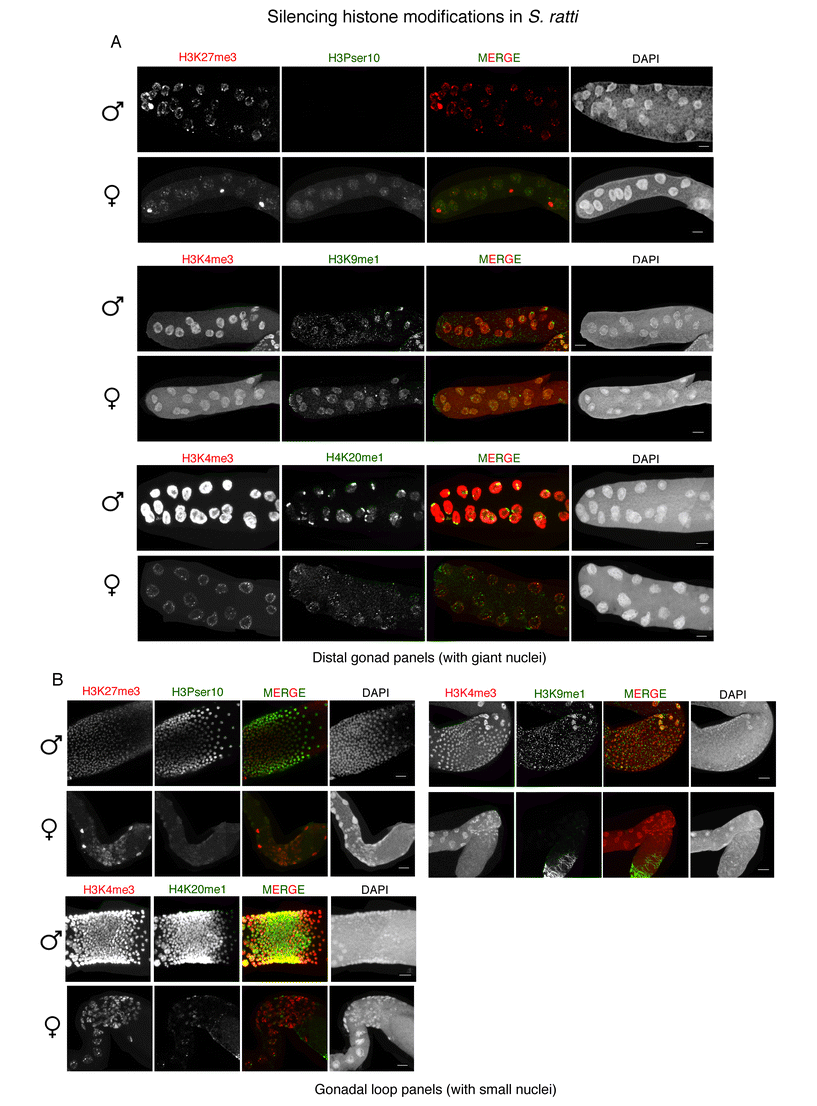

Supplement: Supplementary file 11 — Histone modifications marking silencing in the S. ratti germ line. A. Distal gonad panels from dissected male and female gonads stained against H3K27me3 (top 2 panels), H3K9me1 (middle 2 panels) and H4K20me1 (bottom 2 panels) in combination with H3Pser10 or H3K4me3 respectively. B. Corresponding gonadal loop panels in these males and females for these same antibodies. Scale bar 10 μm. (GIF 218 kb) [file 412_2015_562_Fig15_ESM.gif]
